# Mapping atherogenesis mechanisms in smooth muscle cells by targeting genes linked to coronary artery disease

**DOI:** 10.1016/j.isci.2025.113698

**Published:** 2025-10-04

**Authors:** Julián Albarrán-Juárez, Anton Markov, Anne Louise Jensen, Peter Loof Møller, Anna Katarzyna Uryga, Djordje Djordjevic, Jakob Hansen, Lise Filt Jensen, Diana Sharysh, Charles Pyke, Jaime Moreno, Giulia Borghetti, Julian Bachmann, Kate Herum, Lisa Maria Røge, Matthew Traylor, Michael Nyberg, Mette Nyegaard, Jacob Fog Bentzon

**Affiliations:** 1 Department of Clinical Medicine, Aarhus University, 8200 Aarhus, Denmark; 2 Department of Biomedicine, Aarhus University, 8000 Aarhus, Denmark; 3 Department of Health Science and Technology, Aalborg University, 9000 Aalborg, Denmark; 4 Department of Cardiovascular Biology, Research & Early Development, Novo Nordisk A/S, 2760 Copenhagen, Denmark; 5 Department of Computational Biology, Novo Nordisk A/S, 2760 Copenhagen, Denmark; 6 Department of Genetics, Novo Nordisk A/S, Research Centre Oxford, OX3 7FZ Oxford, UK; 7 Centro Nacional de Investigaciones Cardiovasculares (CNIC), 28029 Madrid, Spain; 8 Steno Diabetes Center Aarhus, Aarhus University Hospital, 8200 Aarhus, Denmark

**Keywords:** cardiovascular medicine, genomics

## Abstract

Genome-wide association studies (GWASs) have linked numerous genetic loci active in vascular cells to coronary artery disease (CAD), implicating smooth muscle cells (SMCs) and SMC-derived mesenchymal cells as potential mediators. We combined CAD GWAS with single-cell RNA sequencing (scRNA-seq) from human atherosclerotic plaques to identify 20 risk genes with putative action in SMCs, and then performed *in vitro* perturbation experiments in SMCs driven toward plaque-relevant phenotypes. Although the genes encode diverse proteins, their perturbations converged on shared transcriptional programs regulating contractile machinery, cell-cycle progression, nuclear factor κB (NF-κB), and type I interferon signaling. Integrating GWAS effect-direction with cholesterol- and stretch-responsive gene modules suggest that cholesterol-induced signaling promotes pro-atherogenic SMC states and is differentially modulated by risk versus protective variants. These results delineate polygenic regulation of SMC disease mechanisms and show that GWAS effect-directionality can help prioritize cellular pathways for follow-up functional studies across genes and cell types.

## Highlights

• GWAS and scRNA-seq highlighted CAD risk genes expressed in human smooth muscle cells
• Risk-gene perturbations showed shared control of contractile, cell cycle, NF-κB, and interferon
• Risk-associated gene variants strengthen cholesterol-induced signaling
• GWAS effect direction predicts which SMC pathways drive CAD and guides therapeutic targeting

## Graphical abstract

**Figure:**
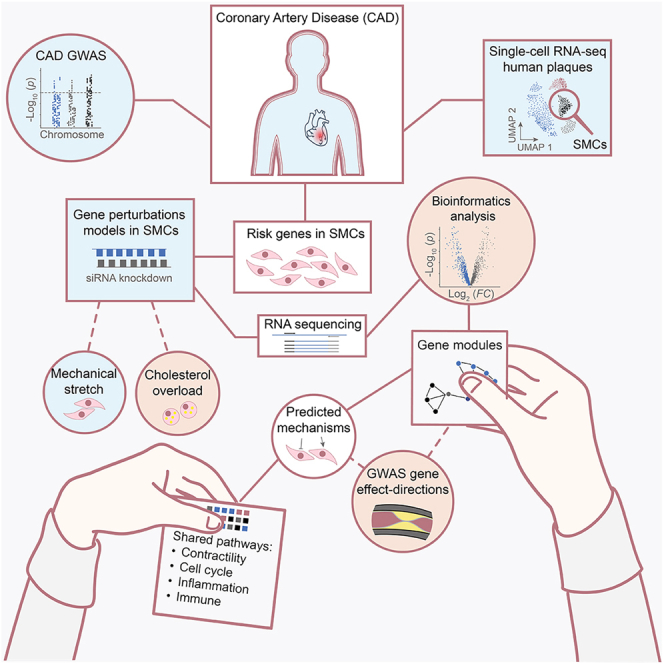

## Introduction

Atherosclerotic cardiovascular disease encompasses myocardial infarction (MI), ischemic stroke, and peripheral artery disease and is the leading cause of death and disability in the world.^1^^,^^2^ Atherosclerosis is caused by high levels of low-density lipoproteins (LDLs) and other risk factors (e.g., hypertension and diabetes), which induce inflammation, activation of local arterial smooth muscle cells (SMCs), and the buildup of fibrous, calcified, and necrotic tissue in the arterial intima. After decades of silent development, plaques may suddenly precipitate thrombosis, leading to clinical disease.^3^ Lifestyle changes and pharmacotherapies that halt disease progression by lowering the causal risk factors for atherosclerosis are the central therapies. Despite the availability of these effective interventions, a significant residual risk remains, thus highlighting the need for original approaches that target different mechanisms.

Vascular SMCs are promising cell targets for alternative therapies that can work orthogonally to risk factor reduction. Recent murine lineage tracing and human single-cell RNA sequencing (scRNA-seq) studies have shown that local SMCs lose their quiescent contractile phenotype, undergo proliferation and modulate to abundant fibroblast-like and osteochondrocyte-like cells during atherogenesis.^4^^,^^5^^,^^6^^,^^7^^,^^8^ Many genes linked to coronary artery disease (CAD) and MI in genome-wide association studies (GWAS) are expressed in SMCs or SMC-derived cells.^9^^,^^10^ This indicates that the expansion, modulation, and function of these cells are critical determinants of the outcome of human atherosclerosis.^11^^,^^12^^,^^13^ The disease-promoting pathways in SMCs are, however, poorly understood, and this impedes their use as targets for drug development.

In this study, we combined public GWAS for CAD and scRNA-seq plaque data to identify risk genes with a putative mechanism of action in SMCs or SMC-derived cells. Rather than analyzing the function of individual risk genes, our approach focused on investigating multiple targets within the same cellular system to uncover shared and functionally significant mechanisms in SMCs. By systematically knocking down 20 risk genes encoding very different types of proteins, we conducted an unbiased analysis of SMC pathways genetically linked to human atherosclerosis, thereby providing insight into potential targets for SMC-directed therapies.

## Results

### Identification of GWAS genes with a potential mechanism of action in SMCs

**Figure 1:**
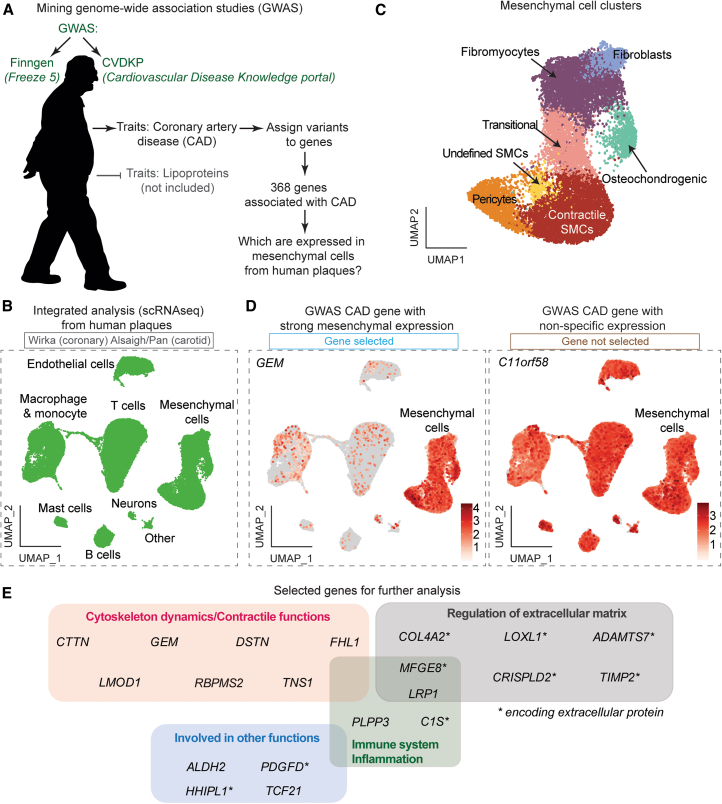
From GWAS and scRNA-seq data to the selection of CAD genes in SMCs (A) GWAS results from Finngen (Freeze 5) and Cardiovascular Disease Knowledge Portal (CVDKP) were utilized to identify variants associated with coronary artery disease (CAD), excluding those associated with lipid traits. After gene fine-mapping, we identified 368 genes. (B) We then tested the expression of these genes on integrated single-cell transcriptomic data from three publicly available studies of human atherosclerotic lesions (in total, 50390 cells from 10 patients).^5^^,^^6^^,^^7^ (C) A supercluster of mesenchymal cells subdivided into 7 clusters that were identified by their marker genes as contractile smooth muscle cells (SMCs), transitional, osteochondrogenic, and undefined SMCs, pericytes, fibromyocytes, and fibroblasts within the integrated data. (D) Examples of genes with enriched expression in mesenchymal clusters (*GEM*) compared to other cells in plaques (*C11orf58*). This analysis led to identifying 45 protein-coding genes preferentially expressed in mesenchymal clusters. (E) Diagram shows the 20 genes selected for further analysis in this study and their putative function. See also Figures S1 and S2, and Tables S1, S2, and S3.

Genomic variants associated with CAD independently of LDL cholesterol were identified in the Cardiovascular Disease Knowledge Portal (CVDKP)^14^ and FinnGen (Freeze 5).^15^ A crude list of 368 candidate genes putatively regulated by these variants was compiled using multiple variant-to-gene mapping strategies in the Open Targets Genetics platform^16^ (Figure 1A; Table S1). To assess which of these genes may have a function in plaque SMCs or SMC-derived cells, we examined their expression in human plaque scRNA-seq data, assembled by integrating published datasets of human coronary and carotid atherosclerosis (Figure 1B; Table S2).^5^^,^^6^^,^^7^ The integrated dataset comprised 50,390 cells after quality control and filtering, including a large mesenchymal supercluster (15,018 cells) encompassing cells with contractile, fibroblast, pericyte, transitional, fibromyocyte, and osteochondrogenic phenotypes, as defined by marker gene expression (Figure 1C). Among the candidate genes, 45 were identified to have increased expression in the mesenchymal supercluster (SMCs, pericytes, and fibroblasts) compared with non-mesenchymal cells (Table S1). To prioritize a manageable subset for further analysis, we chose 16 genes that represented a wide range of putative functions, including 13 with increased expression in mesenchymal cells in both coronary and carotid atherosclerosis (*ALDH2*, *C1S*, *COL4A2*, *CTTN*, *DSTN*, *FHL1*, *GEM*, *LMOD1*, *LOXL1*, *LRP1*, *MFGE8*, *PLPP3*, and *TIMP2*), and a few that were detected as markers in coronary (*CRISPLD2*) or carotid (*PDGFD* and *TNS1*) plaques alone (Figure 1D; Table S1). To this list, we added two genes from previous CAD GWAS studies (*RBPMS2* and *TCF21*) and two genes with rare-mutation evidence in the FinnGen database (*HHIPL1* and *ADAMTS7*), which had supporting evidence for SMC-mediated function based on previous studies,^5^^,^^11^^,^^17^^,^^18^^,^^19^^,^^20^ but were not detected in our pipeline for various reasons, e.g., low expression in scRNA-seq data. The final set of 20 targets (Figure 1E) included both genes with limited prior investigation as CAD GWAS genes (e.g., *C1S*, *CRISPLD2*, *CTTN*, and *GEM*), as well as genes with established roles in plaque SMCs or SMC-derived cells (e.g., *LMOD1*, *MFGE8*, *TCF21*, and *PDGFD*).^21^^,^^22^^,^^23^^,^^24^ The panel was designed in this way to support an investigation of potential shared SMC pathways regulated by multiple risk genes. Lead genomic variants and plaque single-cell expression profiles for the 20 selected genes are shown in Table S3 and Figure S1, respectively.

Expression of the selected target genes was also investigated in a spatial transcriptomics dataset of human coronary plaques.^25^^,^^26^ *MFGE8*, *TIMP2*, *TNS1*, *C1S*, *COL4A2*, *DSTN*, *LMOD1*, and *LRP1* were abundantly expressed in coronary artery plaque and underlying media. *ALDH2*, *CTTN*, *GEM*, *LOXL1*, *PLPP3*, and *RBPMS2* expression were restricted mainly to the media, while *CRISPLD2*, *TCF21*, *HHIPL1*, *PDGFD*, and *ADAMTS7* did not have sufficient detectable expression to determine the localization (Figure S2). *FHL1* was not captured in the Visium panel and therefore no expression data is shown.

### Establishment of cellular assays to evaluate human smooth muscle cell function

**Figure 2:**
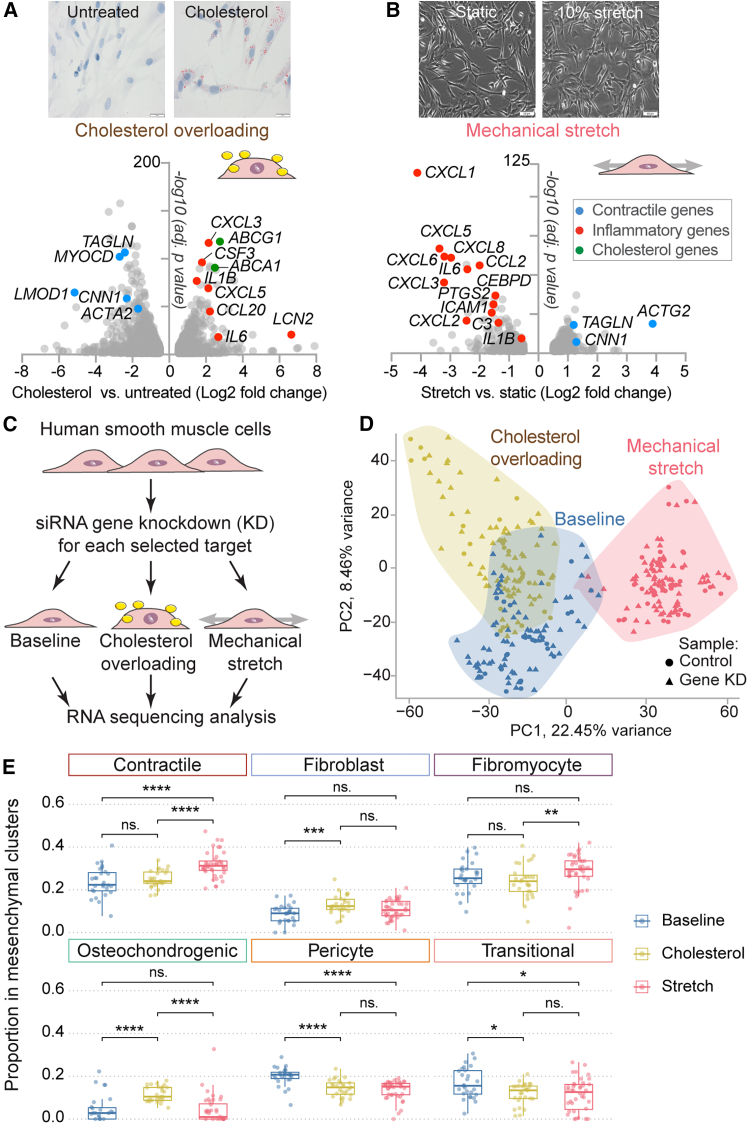
*In vitro* models of vascular disease in human smooth muscle cells (A) Representative brightfield images of untreated human SMCs or after cholesterol overloading stained with Oil red O (ORO) + hematoxylin and eosin. Scale bars: 20 μm. Gene expression comparing cholesterol overload versus untreated SMCs is depicted in the volcano plot. (B) Representative brightfield images of SMCs under static conditions or after physiological (10%, 1 hz, for 6 h) mechanical stretch. Scale bars: 100 μm. Gene expression comparing mechanical stretch versus static SMCs is depicted on the volcano plot. (A and B) show significantly regulated genes (*p* < 0.05 and absolute log2 fold change ≥ 0.5) compared to their control condition (3 technical replicates in each group). DESeq2 was used for data normalization. (C) Schematic of the workflow. SMCs were transfected with siRNAs (pool of two siRNAs) for each selected gene, subjected to different cellular assays, and analyzed by RNA-seq, 3 or 4 technical replicates per gene per assay were included. (D) Principal-component analysis (PCA) of transcriptome profiles of studied SMC samples (*n* = 345) revealed differences between mechanical stretch and other cellular assays. (E) Cell state/subtype deconvolution analysis in cellular assays. Bulk RNA-seq data from SMCs transfected with control siRNA during baseline (*n* = 27), cholesterol overload (*n* = 27), and stretch (*n* = 40) conditions were decomposed into components corresponding to mesenchymal cell clusters identified in single-cell RNA-seq data from three publicly available studies of human atherosclerotic lesions.^5^^,^^6^^,^^7^ A small undefined cell type cluster was excluded from this analysis. Note: Cell-type predictions reflect transcriptomic similarity and do not imply the physical presence of non-SMC cell types in culture. Data are presented as mean ± SEM. *p* values are shown according to the Wilcoxon-Mann-Whitney test and adjustment for multiple comparisons (Bonferroni-Holm method). ∗Adjusted *p* value (*p*adj) < 0.05, ∗∗*p*adj < 0.01, ∗∗∗*p*adj < 0.001, and ∗∗∗∗*p*adj < 0.0001 or ns (not significant). See also Figures S3–S5, and Tables S4 and S5.

To test gene function in several SMC phenotypes relevant to atherosclerotic plaques, we subjected human aortic SMCs to three conditions: baseline, cholesterol overload, and mechanical stretch. The underlying rationale was that target genes that might drive important functions in one context may be inactive in another. The baseline condition was a standard SMC culture, where cells spontaneously modulate, losing contractile proteins, increasing proliferation, and displaying a less elongated morphology.^27^^,^^28^ The cholesterol overload condition was achieved by treating SMCs with water-soluble cholesterol to drive cholesteryl ester droplet formation in SMCs,^29^ which leads to a foam cell-like state characterized by increased inflammatory signaling.^30^^,^^31^ RNA sequencing (RNA-seq) analysis confirmed that 72 h of cholesterol overloading in SMCs increased lipid accumulation and upregulated inflammatory genes (e.g., *CCL20*, *CXCL2*, *CXCL3*, *CXCL5*, and *IL1B*), cholesterol efflux genes (*ABCA1* and *ABCG1*) and downregulated contractile genes (e.g., *ACTA2*, *CNN1*, *LMOD1*, *MYOCD*, and *TAGLN*) compared with cells cultured under control conditions (Figure 2A). Finally, in the mechanical stretch condition, physiological mechanical forces (10% elongation) were applied to potentially drive SMCs toward a less inflammatory and more contractile phenotype.^32^ Interestingly, 6 h of physiological mechanical stretching downregulated several inflammatory genes (e.g., *CCL2*, *CXCL1*, *CXCL3*, *IL1B*, and *IL6*) and upregulated contractile genes (e.g., *CNN1*, *ACTA2*, *TAGLN*, and *ACTG2*, but not *LMOD1*) compared with SMCs cultured similarly under static conditions (Figure 2B). The complete gene expression results comparing cholesterol overload or stretch versus untreated SMCs are shown in Table S4.

### Knockdown of genes associated with coronary artery disease in human SMCs

To analyze the function of the selected 20 target genes in human SMCs, we knocked down each of them by custom-designed small interfering RNAs (siRNAs) and subjected cells to baseline, cholesterol overload, or mechanical stretch conditions. Effects on gene expression were analyzed by RNA-seq using a nested design where 2–3 target gene knockdowns were run concurrently with control siRNA-treated cells to mitigate batch effects (Figure 2C). Overall, we analyzed the transcriptome of 345 samples (3–4 technical replicates per target gene and condition). The numbers of differentially expressed genes (DEGs) compared with controls were higher under baseline conditions (from 159 to 3,266 DEGs, 884 at average) and cholesterol loading (from 107 to 3,122 DEGs, 1,078 at average) than after mechanical stretch (from 12 to 1,612 DEGs, 592 at average). All data generated by RNA-seq for all conditions and samples is deposited at Zenodo (see data and code availability). Principal-component analysis (PCA) of total RNA-seq results confirmed the transcriptional changes across the baseline, cholesterol overloading, and mechanical stretch conditions (Figure 2D). It also revealed considerable overlap between baseline and cholesterol loading conditions.

To determine how the *in vitro* conditions in our experiments reflect relevant mesenchymal cell states observed in atherosclerotic lesions *in vivo*, we performed deconvolution of our bulk RNA-seq data from the experimental samples using gene expression patterns from mesenchymal cell clusters identified in the integrated scRNA-seq dataset. Analysis of SMCs transfected with control siRNA predicted more contractile phenotypes in response to mechanical stretch, more osteochondrogenic phenotypes among cholesterol-overloaded SMCs, and a greater prevalence of transitional phenotypes under baseline conditions (Figure 2E; Figure S3). These predicted cell-type signatures reflect transcriptional similarity between modulated SMCs and mesenchymal phenotypes observed *in vivo*, rather than the presence of distinct cell types in culture. Nevertheless, these findings confirm that our cellular assays induce, at least to some degree, cellular states that are relevant to those observed in atherosclerotic lesions *in vivo*. The absolute expression levels (TPM) of the candidate genes at baseline (control siRNA and after target siRNA knockdown) are shown in Table S5. The knockdown efficiency of the target genes was also analyzed by quantitative real-time PCR (real-time qPCR), as shown in Figure S4. Gene knockdown analysis was efficient for most target genes, decreasing the level of target gene expression by 75%–90% compared to control samples. However, for *TCF21* and *PLPP3*, the designed siRNAs reduced expression by less than 50%. Therefore, *TCF21* and *PLPP3* were not further analyzed in the study. RNA-seq data confirmed the knockdown efficiency and, interestingly, showed mutual regulation among the target genes (Figure S5). For example, the knockdown of *LMOD1* significantly upregulated the expression of *GEM*, *CTTN*, and *TNS1* and downregulated the expression of *ALDH2*, *COL4A2*, *CRISPLD2*, *FHL1*, *PDGFD*, and *TIMP2* in baseline and cholesterol overload conditions.

### Regulation of smooth muscle cell pathways after target gene knockdown

**Figure 3:**
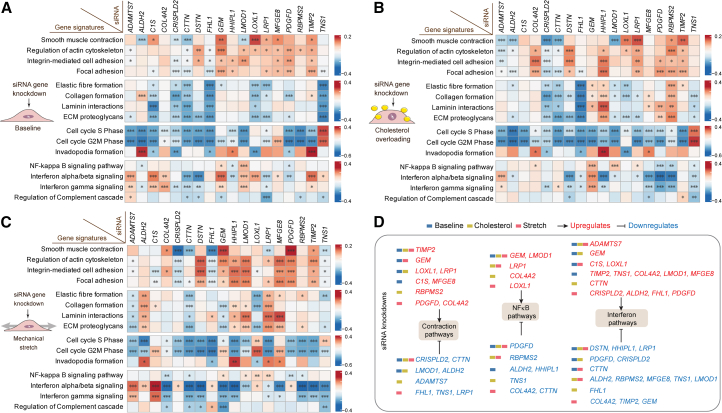
Gene signatures disturbed after target gene knockdown (A–C) Expression-based scoring of gene signatures implicated in SMC contractility, synthesis of extracellular matrix proteins, cell cycle, and inflammatory response in the different cellular assays: (A) baseline, (B) cholesterol overload, and (C) mechanical stretch. Specific gene sets were collected from KEGG Pathway, Reactome, and WikiPathways as shown in Table S6. The color scale represents a *Z* score of deviation in the transcriptional activity of relevant pathway genes in target knockdown compared with a control group (3 or 4 technical replicates in each group). Asterisks highlight statistical significance based on false discovery rate (FDR)-adjusted *p* values (*p*adj): ∗*p*adj < 0.1, ∗∗*p*adj < 0.01, and ∗∗∗*p*adj < 0.001. ECM, extracellular matrix. (D) Examples of the most regulated pathways by differentially expressed genes induced by siRNA knockdowns of target genes. See also Figure S6 and Tables S6, S7, and S8.

Gene set enrichment analysis (GSEA) for all comparisons in this study showed that cell cycle, interferon signaling, inflammatory cytokines, and Rho GTPases were among the predominant (normalized enrichment score) gene ontology categories and biological pathways regulated after target gene knockdowns (Table S6). For a more direct analysis of pathways and genes relevant to SMC functions, we analyzed tailored gene signatures representing SMC contraction, cytoskeleton, cell adhesion, extracellular matrix organization, cell cycle, and inflammatory/immune responses, collected from several sources, including Kyoto Encyclopedia of Genes and Genomes (KEGG),^33^ Reactome,^34^ and WikiPathways^35^ (Table S7). Gene sets involved in SMC contraction, extracellular matrix synthesis, and cell cycle were regulated by multiple target genes, and we also identified a strong regulation of the nuclear factor kappa-light-chain-enhancer of activated B cells (NF-κB) and interferon pathways (Figures 3A–3C).

For a subset of target genes (e.g., *ADAMTS7*, *CTTN*, *GEM*, *LMOD1*, *PDGFD*, and *TIMP2*), the regulation of specific signaling pathways remained consistent across all examined cellular states, but often, it was only detected in one or two of the examined cell states and, in some cases (e.g., *LRP1*, *COL4A2*, and *TNS1*), in opposite directions, as exemplified for contraction, NF-κB, and interferon signaling in Figure 3D. This may indicate that certain mechanisms gain or wane in importance within different cell states. For example, genes involved in extracellular matrix organization were generally downregulated by knockdowns in the baseline conditions, presumably reflecting the high activity of these genes in this cell state, while up- and downregulation were seen in other states (Figures 3A–3C). Basal expression of the target genes, although in several cases up- or downregulated by cholesterol or stretch (Figure S6), did not predict knockdown outcomes.

To validate the observed regulation of NF-κB- and type I interferon-induced genes, we performed two types of experiments. First, we tested whether the siRNA transfections themselves could cause the observed inflammatory signaling. Depending on specific recognition motifs in the sequence, some siRNAs can directly activate type I interferon responses.^36^^,^^37^ The siRNAs used in our experiments were designed by the manufacturer QIAGEN to not contain these motifs, and we confirmed experimentally that neither the control siRNA nor lipofectamine transfection alone altered the expression of *IL6*, *IFI6*, *IFITM1*, or the contractile marker *MYOCD* compared with untreated controls (Figure S7).

Second, since genes induced by NF-κB and type I interferon signaling may vary by cell type, we defined the NF-κB and type I interferon gene signatures in human SMCs by stimulating with the prototypical inducers tumor necrosis factor (TNF) and interferon alpha (IFN-α) followed by RNA-seq (Figures S8A and S8B; Table S8). We found that the experimentally defined gene sets were partly overlapping and confirmed that they were regulated by multiple target genes with *C1S* and *HHIPL1* knockdown, producing the strongest up- and downregulation across the three cell states (Figure S8C).

### Widespread alterations in inflammation- and cell cycle-related regulons

**Figure 4:**
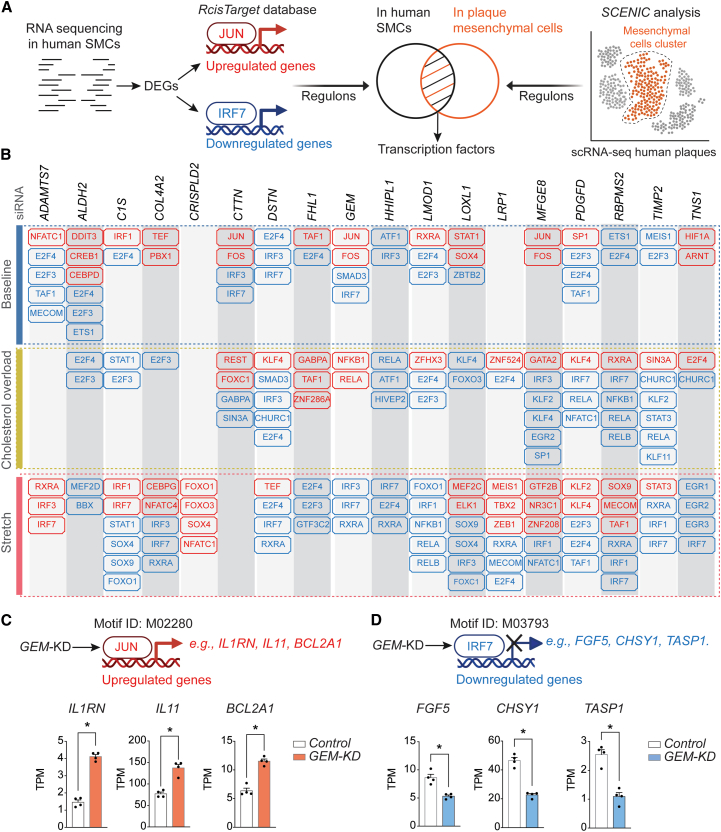
Regulons with altered activity following target knockdown within the different cellular assays (A) Regulon is a collection of genes with a shared transcription factor (TF) binding site suggesting potential co-regulatory mechanisms by identical TFs. For each targeted gene knockdown and corresponding cellular assay in human SMCs, we identified regulons with a significant presence within the differentially expressed genes (DEGs), separately for up- and downregulated gene sets (JUN and IRF7 are shown as examples). Identified regulons were then overlapped with regulons detected to be active in at least one mesenchymal subcluster by SCENIC analysis of the integrated scRNA-seq dataset from three publicly available studies of human atherosclerotic lesions. (B) Shown are examples of shared regulons across various mesenchymal cell clusters altered by targeted gene knockdown in cellular assays named by putatively binding TFs. A complete list of regulons and their enriched genes is available in Table S9. Regulons with up- and downregulated genes are depicted as red and blue boxes, respectively. (C and D) Regulation of JUN or IRF7-dependent genes by GEM knockdown (KD) is shown as an example. Control and GEM-KD samples (four technical replicates per group) were compared by Mann-Whitney test. ∗Adjusted *p* value (*p*adj) < 0.05. Data are presented as mean ± SEM. Schematics made with Biorender.com. See also Table S9.

To identify the transcription factors (TFs) that drive the shared transcriptomic changes among different knockdowns, we detected regulons (a set of genes jointly regulated by the same TF) among up and downregulated genes. To focus on regulons with *in vivo* relevance, we restricted our analysis to regulons that were detected to be active (high *Z* score) in at least one mesenchymal cell subcluster of human plaques by scRNA-seq analysis with SCENIC (Figure 4A). Corroborating our analysis on the pathway and marker gene levels, we found that regulons putatively controlled by cell cycle/proliferation (E2F3, E2F4, ETS1, NFATC1, SP1, MEIS1, and TAF1) and immune/NF-κB (IRFs, STAT, RELA, RELB, and NFKB1) TFs were significantly enriched. Members of the AP-1 complex (FOS and JUN), SOX, and Kruppel-like factors known as regulators of SMC phenotype were also identified (Figure 4B). A complete list of the regulons and enriched genes identified in every targeted gene knockdown, across the different cellular assays, including TNF and IFN stimulation, is shown in Table S9. As an example, we examined the absolute expression levels of target genes from regulons affected by *GEM* knockdown, such as JUN and IRF7. While JUN-target genes (e.g., *IL1RN*, *IL11*, and *BCL2A1*) are increased upon GEM knockdown (Figure 4C), IRF7-dependent genes (e.g., *FGF5*, *CHSY1*, and *TASP1*) are reduced compared to control samples (Figure 4D).

### Independent regulation of common SMC marker genes and functional readouts

The growth of SMC-derived cells in atherosclerosis involves loss of SMC contractile gene expression, increased proliferation, and switching to modulated cell phenotypes characterized by extracellular matrix production and inflammatory signaling.^8^^,^^38^^,^^39^ This metaplastic process explains the inverse correlation between contractile genes and markers of modulated cells and inflammatory signaling in plaque scRNA-seq data.^8^ It is, however, less clear if reciprocal regulation of these programs already exists within a particular SMC cell state. Future studies employing single-cell transcriptomic profiling under these conditions will be necessary to definitively determine whether these programs are mutually regulated at the single-cell level. Since our goal was to examine general trends in expression profiles consistent with known SMC phenotypic switching, we retrieved the expression levels of multiple marker genes used in the literature to define SMC phenotype. We including contractile gene markers (*ACTA2*, *ACTG2*, *CNN1*, *LMOD1*, *MYOCD*, and *TAGLN*), modulated SMC markers (*FN1*, *KLF4*, *LUM*, and *SPP1*), cell proliferation markers (*MKI67* and *PCNA*), NF-κB activation markers (*IL1B*, *IL6*, *PTGS2*, and *VCAM1*), and type I interferon-induced genes (*IFIT1*, *IFITM1*, *ISG15*, *MX1*, and *OAS1*).

**Figure 5:**
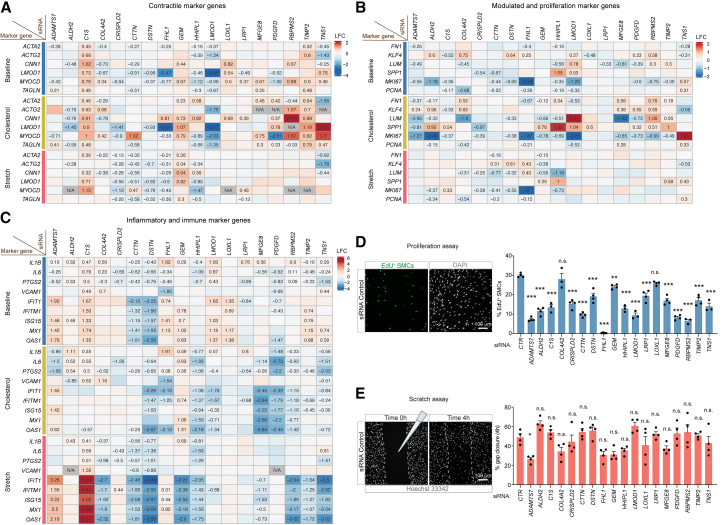
Regulation of conventional gene markers for smooth muscle cell function (A–C) Differences in the expression of a subset of marker genes important for smooth muscle cell function in target gene knockdowns compared to related control groups on each cellular assay. Results are shown for different cellular pathways: (A) contractile, (B) modulated and proliferation, and (C) inflammatory and immune marker genes. Numbers indicate log2-transformed fold changes (LFC), shown only statistically significant gene regulation (*p*adj < 0.05. N/A indicates that gene expression was not detected. (D) SMCs were transfected with negative siRNA (control) or a pool of two siRNAs targeting each gene as described and labeled with EdU (5-ethynyl-2′-deoxyuridine) for 24 h to evaluate their proliferation. Shown is a representative image of the technique and the percentage of EdU+ SMCs per view field (3–4 technical replicates were evaluated per gene). (E) SMCs were transfected with the different siRNAs, and one day later a scratch was made in the center of the well and followed with a time-lapse microscope to evaluate their migration rate (representative images are shown). Cell nuclei were labeled with Hoechst 33342 live staining (NucBlue) and incubated at 37°C and 5% CO_2_ in a time-lapse microscope (Nikon, Eclipse Ti2). Cell migration was monitored for 24 h in total. The percentage of gap area closure is shown 4 h after scratch (3–4 technical replicates were evaluated per gene). Samples in D and E were compared to the control cells using a one-way ANOVA followed by a Dunnett test. Data are presented as mean ± SEM. Asterisk ∗ represents an adjusted *p* value (*p*adj) < 0.05, ∗∗*p*adj < 0.01, and ∗∗∗*p*adj < 0.001, or n.s. (not significant, *p* value >0.05) compared to control.

Contractile marker gene expression was widely regulated under baseline conditions, being stimulated by *C1S*, *GEM*, *LOXL1*, *MFGE8*, *RBPMS2*, and *TIMP2* knockdown and repressed by *ADAMTS7*, *ALDH2*, *COL4A2*, *CRISPLD2*, *CTTN*, *DSTN*, *FHL1*, *HHIPL1*, *LMOD1*, and *PDGFD* knockdown (Figure 5A). Changes in cholesterol-overloaded and stretched cells were similar, albeit with some differences in the magnitude of regulation. The selected contractile genes were mostly regulated as a group, consistent with their common transcriptional regulation,^40^ with *MYOCD* showing the most independent regulation.

Interestingly, the selected markers for modulated SMCs (*FN1*, *KLF4*, and *LUM*) were often independently regulated and not necessarily upregulated when contractile genes were repressed and vice versa. (Figure 5B). Neither did we observe a consistent reciprocal regulation between contractile genes and marker genes for proliferation (*MKI67* and *PCNA)*, which were downregulated after all target gene knockdowns except for *TNS1* (Figure 5B). Moreover, while markers for NF-κB signaling and type 1 interferon signaling were regulated by multiple target genes (Figure 5C), those knockdowns that dampened inflammatory signaling, e.g., *DSTN* and *CTTN*, were not necessarily the same that upregulated modulated SMC markers and vice versa.

Finally, we examined proliferation and migration, which are functional readouts often used to gauge SMC phenotype. Proliferation measured by EdU labeling was downregulated in almost all target gene knockdowns (except for *COL4A2* and *LOXL1*, which showed no difference), consistent with the cell cycle marker gene analysis (Figure 5D). In contrast, a significant difference in cell migration (scratch assay) was only detected for *ADAMTS7* knockdown (Figure 5E).

Overall, this analysis, made possible by having a large set of independent cell perturbations, indicated that contractile gene expression and markers/functional readouts of proliferation, modulation, and inflammatory signaling are not tightly co-dependent in cultured SMCs. This is important because such markers are often used as markers for SMC phenotypic switching in cell culture under the assumption that they are linked and report cell identity changes similar to those occurring during atherosclerosis, which appears not to be justified.

### Proatherogenic effect of cholesterol overload indicated by GWAS gene effect directions

The genetic variation identified in GWAS studies mostly influences CAD risk by altering gene expression rather than through alterations in the encoded protein, and genes can be detrimental or protective depending on whether lower expression decreases or increases disease risk, respectively. To classify our selected target genes, we identified co-localizing expression or protein quantitative trait loci (e/pQTLs) and determined their impact on CAD risk by Mendelian randomization.^41^ We used eQTL data from GTEx V8^42^ and eQTLgen,^43^ and pQTL data from UK Biobank^44^ and a large Icelandic cohort.^45^

The analysis predicted *ALDH2*, *C1S*, *CTTN*, *LOXL1*, *MFGE8*, *PDGFD*, and *TIMP2* to be detrimental and *FHL1*, *HHIPL1*, *LMOD1*, *LRP1*, and *RBPMS2* to be protective genes for CAD development (Table S10). The analysis was inconclusive for the remaining genes.

We then used this classification as a tool to estimate which of the many regulated pathways identified in our SMC analysis were likely pro- versus anti-atherogenic. The underlying rationale is that a pro-atherogenic mechanism will tend to be upregulated by the knockdown of protective genes, while an anti-atherogenic mechanism will tend to be upregulated by the knockdown of detrimental genes.

**Figure 6:**
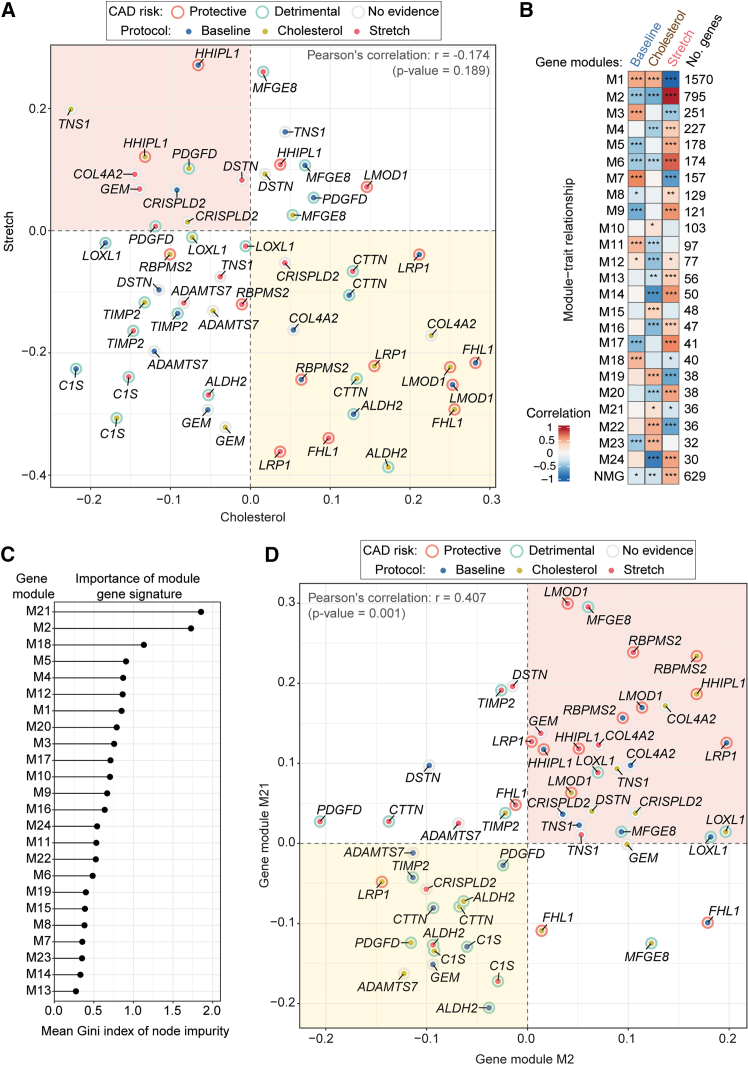
Gene signatures in human smooth muscle cells (A) Regulation of cholesterol and stretch-specific gene signatures by individual gene knockdowns. The *x* and *y* axes represent the degree to which each gene knockdown upregulates or downregulates the top 50 genes uniquely induced by cholesterol overload or mechanical stretch, respectively (i.e., signature scores). Each point corresponds to a target gene knockdown. The color of the circle indicates the inferred direction of effect on CAD risk based on e/pQTL colocalization and Mendelian randomization (salmon = protective, emerald = detrimental, and gray = no evidence). The type of *in vitro* condition in which the knockdown was profiled (baseline, cholesterol, or stretch) is indicated by dot fill pattern. This analysis is only hypothesis-generating and explores whether SMC signatures induced by cholesterol or stretch are differentially regulated by protective versus detrimental CAD genes. (B) Correlation of co-expressed gene modules with cellular assays. The color scale on the heatmap represents the Pearson’s correlation coefficient between module eigengene (summarized expression profile) and coaxed cellular state. Asterisks highlight significant correlation: ∗*p* < 0.05, ∗∗*p* < 0.01, and ∗∗∗*p*< 0.001. NMG, non-module genes not clustered in any co-expressed module. (C) Random forest model and Gini index-based estimate of feature importance were used to prioritize identified module gene signature scores by their ability to discriminate between target genes with detrimental and protective effects on CAD. The higher the estimate, the better the gene module score predicts the opposite effects of target genes. (D) Relationship between the top gene modules (M2 and M21) gene signature dynamics in target gene knockdowns, genetic effect direction of target genes, and cellular assay. The top 100 highly connected genes were used in the analysis for the large M2 module. See also Figures S9 and S10, and Tables S10, S11, and S12.

Analyzing the broader gene signatures induced by cholesterol overloading and stretch revealed that cholesterol overloading in SMCs had the characteristics of a pro-inflammatory mechanism (Figure 6A): knockdown of protective genes, such as *LMOD1*, *FHL1*, or *LRP1*, upregulated the cholesterol overload-induced gene signature, while knockdown of detrimental genes, such as *C1S* and *TIMP2*, downregulated it. Interestingly, this pattern of opposite regulation by protective and detrimental genes was not seen among gene knockdowns that simultaneously upregulated stretch-induced genes, suggesting some level of interaction (e.g., *HHIPL1* and *MFGE8*).

Individual pathways (e.g., SMC contractility, NF-κB signaling, and type I interferon) were not consistently regulated by protective versus detrimental genes (analyses not shown). This may reflect the complex and mutually independent regulation of these pathways across the gene knockdowns, necessitating a multivariate analysis to disentangle that was not possible with our limited number of informative genes.

### Specific SMC gene modules regulated by protective and detrimental GWAS genes

To evaluate which parts of the cholesterol- and stretch-induced responses may be pro- or anti-atherogenic, we defined gene modules in SMCs subjected to baseline, cholesterol overload, and mechanical stretch conditions by analyzing gene co-expression in the 87 samples transfected with control siRNA. We obtained 24 modules (M1–M24), ranging in size from 1,570 genes in M1 to 30 genes in M24, with evidence of differential regulation across the SMC states (Figure 6B). Details of genes and their connectivity are provided in Table S11, and a graphical representation of the analysis can be found in Figure S9.

We then explored which of these 24 gene modules meets the criteria of a pro- or anti-atherogenic mechanism, being regulated oppositely (if at all) by protective and detrimental genes. Gene module 2 (M2) and module 21 (M21) were the top modules identified with these characteristics (Figure 6C). M2 (795 genes) was characterized by genes with high activity in the stretch condition but low in baseline and cholesterol-overload conditions. In comparison, M21 (36 genes) contained genes with high activity during cholesterol-overload conditions but not at baseline or stretch. Enrichment analysis revealed that M2 contained genes involved in RNA processing, while M21 comprised genes associated with the wingless-type MMTV integration site family (Wnt) signaling pathway (e.g., *WNT7B*, *WNT5B*, *FZD1*, *TCF7*, among others) (Table S12). Expression of the two modules across the target knockdowns showed a positive correlation, and both had the signature of a pro-atherogenic mechanism being upregulated by protective gene knockdowns (e.g., *LMOD1* and *HHIPL1*) and downregulated by detrimental gene knockdowns (e.g., *ALDH2* and *C1S*) (Figure 6D). These elements of SMC responses to metabolic and mechanical stress may, therefore, be particularly interesting for further exploration. An expression-based gene scoring analysis for all the modules is shown in Figure S10.

## Discussion

Many CAD susceptibility genes in GWAS studies do not exert their effects through classical risk factors such as circulating cholesterol levels or hypertension.^46^ Therefore, unidentified disease mechanisms contributing to residual risk, which are not addressed by current therapies, may exist. During atherosclerosis, a subset of SMCs de-differentiate, proliferate, and modulate to other mesenchymal cell phenotypes, many of which abundantly accumulate cholesteryl ester lipid droplets.^8^^,^^47^^,^^48^ The accumulation of the SMC-derived cells and the extracellular matrix proteins they secrete is a central mechanism by which atherosclerotic plaque grows and a main determinant of plaque stability.^49^ Cells with SMC-like phenotype build the fibrous cap, which protects against plaque rupture and thrombosis, but the modulated SMCs in the plaque interior may play detrimental roles by contributing to the formation and expansion of the necrotic core.^50^^,^^51^ The phenotypic transition of SMCs and the important functions of the modulated cell types in plaques make them interesting therapeutic targets, but little is known about the molecular pathways that could be targeted.

Recent studies have searched for genes regulating a fixed set of atherosclerosis-relevant SMC functions (e.g., proliferation and inflammatory gene expression) using the genetic variation across a large set of patient-cultured SMCs.^52^^,^^53^ Here, we followed the complementary strategy of introducing gene perturbations experimentally and performing an open search for alterations in SMC gene expression and function. This has previously been done for individual genes, but as exemplified by our findings, many patterns of atherosclerosis-relevant SMC regulation can only be revealed by looking at multiple gene perturbations in the same context. We systematically performed a knockdown screen of genes that are expressed in SMCs or their modulated progeny in coronary and carotid plaque scRNA-seq datasets and have genetic evidence for a causal role in CAD that is not explained by plasma lipids. Spatial transcriptomics of human coronary plaques further supported the relevance of our selected candidate genes, showing that many are expressed in regions corresponding to the plaque and underlying media, consistent with a role in vascular remodeling and disease. The goal was to use these knockdowns as an instrument to understand what gene signatures and pathways are relevant for the pathophysiological function of SMCs in CAD. While it is not yet possible to recreate the SMC phenotypic transitioning process *in vitro*, we steered human SMCs to several plaque-relevant states to increase the likelihood that central pro- or anti-atherogenic mechanisms of SMCs were featured in our assays. Finally, by integrating what is known about the effect of CAD on gene variants controlling the expression of our GWAS target genes, we attempted to determine which of the observed regulated pathways are pro-atherogenic and anti-atherogenic.

Most of the genes targeted in our study have not previously been investigated for their role in human SMC function or modulation (e.g., *C1S*, *CRISPLD2*, *CTTN*, and *GEM*), but some have been examined in independent studies, which can be used for cross-validation of our data.^19^^,^^20^^,^^54^^,^^55^^,^^56^^,^^57^^,^^58^ PDGFD was shown previously to promote the modulation of cultured SMCs and increase inflammatory signaling,^18^ while LMOD1 was required for the maintenance of the contractile phenotype.^21^^,^^22^ Overall, our analysis produced four key findings. Firstly, we observed widespread regulation of cytoskeletal and contractile genes, NF-κB, and type I interferon-induced genes, as well as cell cycle genes by the target gene knockdowns at both the pathway, marker gene, and regulon analysis levels. To assess the CAD relevance of the transcriptional changes induced by our gene knockdowns, we performed GSEA of the knockdown signatures (Table S6). These analyses revealed enrichment of several atherosclerosis-related pathways, supporting the *in vivo* relevance of the observed gene programs. Further studies integrating heritability enrichment methods and direct comparisons to *in vivo* datasets may provide additional insights into the causal role of these programs in human disease. Importantly, there is experimental evidence from mouse models that several of these programs in SMCs are causal mediators of atherogenesis. Heterozygous deletion of MYOCD, the transcriptional co-activator of most contractile genes, results in accelerated atherosclerosis with more inflammatory cells in mice.^59^ NF-κB signaling in cultured SMCs interferes with MYOCD function and drives proliferation,^60^ and knockout of the gene encoding the required signaling factor IKKβ in SMCs protects against the development of murine atherosclerosis.^61^ Furthermore, we recently found that the depletion of modulated SMCs in regressing atherosclerosis is associated with the loss of NF-κB signaling in those cells.^8^ In contrast, the role of type I interferon signaling in SMCs and atherosclerosis is less understood,^62^ but the strong regulation observed here, which could not be attributed to artifacts of siRNA transfection, warrants further studies of the potential causal role. Finally, the observed almost uniform downregulation of cell cycle genes and proliferation rates in cell culture assays was striking (Figures 5B and 5D). SMC proliferation is a key driver of plaque development in atherosclerosis, but it is unlikely that almost all the GWAS genes, including both protective and detrimental ones, regulate proliferation in the same direction *in vivo*. We speculate that our findings can be explained by cultured SMCs being selected for their ability to proliferate—those that proliferate better overgrow others. If the state of the cell is optimized for the fastest possible proliferation, it makes sense that most perturbations to the cell would result in reductions in proliferation. This inherent quality of SMC culture can easily be overlooked when gene knockdowns are examined individually.

A second key finding of our study is that several gene knockdowns produced distinct effects depending on the experimental condition—baseline, cholesterol overload, or mechanical stretch. In many cases, a given knockdown had a significant effect in one context but not in others; in some cases, the effect was even reversed (see Figure 3D). This highlights the critical role of cellular context in shaping gene function and underscores a broader challenge in the field: the lack of consensus on which *in vitro* conditions best reflect *in vivo* SMC behavior.

Thirdly, our results show that functional phenotypes such as migration and proliferation, as well as commonly used SMC marker genes, were regulated independently of one another across these treatment conditions. This suggests that cholesterol loading and mechanical stretch do not uniformly drive the same SMC phenotypic shifts and should not be assumed to predict identical functional outcomes. Independent patterns of SMC marker expression and functions have been reported previously.^63^ This contrasts with the clear inverse associations between contractile gene markers (e.g., *ACTA2* and *MYH11*) and markers of modulated SMCs (e.g., *LUM*) and inflammatory signaling in plaque scRNA-seq data,^5^^,^^8^ and the fact that healthy contractile SMCs are non-proliferative and non-migrating, whereas activated, non-contractile SMCs in disease expand and move. The likely explanation is that studies in cultured SMCs are confined to examining cellular mechanisms in a semi-modulated, proliferative state, whereas SMCs *in vivo* undergo much more pervasive changes, transitioning from contractile SMCs characterized by low NF-κB signaling and minimal synthetic gene expression to non-contractile mesenchymal cell types exhibiting active NF-κB signaling and robust synthetic gene expression. These cellular transitioning processes cannot yet be fully modeled *in vitro*, and none of the knockdowns were consequential enough to transform the overall cell phenotype. This is evidenced by the PCA analysis in Figure 2E, which shows that in a given assay no knockdown shifted gene expression from one cell state cluster to another. While no individual gene knockdown in our study resulted in a full switch between SMC states at the transcriptomic or phenotypic level, we recognize that earlier or more subtle transitions may be epigenetically primed. Future studies incorporating epigenomic profiling will be important to identify chromatin-level changes that may precede and facilitate transcriptional and functional remodeling of SMCs. The key takeaway is to acknowledge this limitation of SMC culture studies, and not use SMC markers, proliferation, and migration *in vitro* as if they reported on the full trajectory that SMCs take in atherosclerotic plaques *in vivo*.

The final key finding was that the cholesterol-induced gene response had characteristics of a pro-atherogenic mechanism. Among the gene knockdowns that regulated cholesterol-induced genes, upregulation was seen after the knockdown of predicted protective genes and downregulation after the knockdown of predicted detrimental genes (Figure 6). This type of analysis is only hypothesis-generating, but, importantly, the causal role of cholesterol overloading in SMCs is supported by experimental evidence. Cholesterol overload of cultured SMCs with cyclodextrin-complexed cholesterol, as performed in this study, leads to foam cell formation with cholesteryl ester droplet accumulation.^29^ While this method may not generate cells that fully acquire the transcriptional profile of SMC-derived foam cells *in vivo*,^31^ it induces transcriptional changes that overlap with gene expression of modulated SMCs in progressing mouse lesions.^8^ Part of this regulation appears to be mediated by cholesterol overload-induced activation of NF-κB signaling,^30^ possibly through eliciting endoplasmic reticulum stress.^64^ It will be important to investigate the upstream and downstream mechanisms of cholesterol overload in SMCs to identify nodes that can be targeted in experimental models to understand causal mechanisms and, potentially, as future targets for therapy. Further explorations of *C1S*, *LMOD1*, *FHL1*, *LRP1*, and *TIMP2*, identified here as strong regulators of the cholesterol-induced gene response, and the specific gene modules M2 and M21 implicated in the mechanism, can be starting points.

In a general perspective, the approach taken here, combining an *in vitro* screen with genetic effect predictions, can serve as proof-of-concept for larger-scale studies in SMCs or other atherosclerosis-relevant cell types to predict the effect of blocking versus stimulating specific mechanisms in atherosclerosis.

In summary, our study maps major mechanisms regulated by a set of CAD risk genes with a likely mechanism of action in plaque SMCs. To illustrate the added value of our study, we would like to highlight GEM as an example of a previously underappreciated gene with potential regulatory roles in SMCs pathways relevant to CAD. GEM was found to modulate contractile, inflammatory, and proliferative pathways in a context-specific manner in SMCs, highlighting its potential role in disease-relevant cellular states.

By integrating knowledge of the effect direction of a subset of the analyzed GWAS genes, we identify cholesterol overload of SMCs, as well as specific cholesterol- and stretch-regulated gene modules, as putative pro-atherogenic mechanisms regulated by human genetic predisposition to CAD. The data released with this report include 345 RNA-seq datasets that can be used to explore the mechanisms downstream of 18 SMC-relevant GWAS genes, the effect of cholesterol overload and mechanical stretching on SMC cell state, and interactions between GWAS gene manipulation and SMC state.

### Limitations of the study

There are several important limitations in our study to consider. First, cultured SMCs, even if subjected to cholesterol overload and stretch, do not mimic the full diversity of SMC-derived cell phenotypes in atherosclerosis. Second, the study used aortic SMCs from a single donor for all experiments. While this reduced variation across individual assays and improved our ability to detect gene knockdowns effects, the use of a single donor may limit the generalizability of the findings, as it does not account for inter-individual genetic variability in SMC responses.^52^ Furthermore, it will be prudent to validate the observed effects in SMCs from other vascular beds. That said, many CAD-associated genes and pathways characterized here have also been implicated in coronary artery SMCs in previous studies. We acknowledge that vascular bed-specific differences exist in SMC gene expression and function. Although our experiments were conducted using aortic SMCs, many CAD-associated genes and pathways characterized here have also been implicated in coronary artery SMCs in previous studies.^18^^,^^19^^,^^21^^,^^53^^,^^54^^,^^55^^,^^56^^,^^57^^,^^58^^,^^65^^,^^66^ Moreover, future functional studies incorporating cytoskeletal and immunophenotypic analyses using coronary artery-derived SMCs will be essential to validate the conservation and specificity of these mechanisms in the context of CAD. Third, we used Mendelian randomization based on e/QTLs to propose a direction of effect between target gene levels and CAD risk. Co-regulation of neighboring genes indicates that *cis*-QTL effects are often non-specific and reflect alterations to multiple nearby genes.^67^ Therefore, Mendelian randomization estimates based on e/QTLs may not reflect transcriptomic changes that are limited to the target gene. Although Mendelian randomization gives an estimate of whether a given gene is causally involved in disease, it does not necessarily tell us through which pathway the disease risk is altered. In this analysis, it is possible that some genes might act through alternate mechanisms. Fourth, the number of informative GWAS genes on our screen was insufficient for fully exploring the relationships between the effect of direction of GWAS genes on human CAD and the mechanisms they regulate in SMCs.

## Resource availability

### Lead contact

Requests for further information and resources should be directed to and will be fulfilled by the lead contact, Julián Albarrán-Juárez (jalbarran@clin.au.dk).

### Materials availability

This study did not generate any new or unique reagents.

### Data and code availability

• All RNA-seq data from siRNA-guided target gene knockdown experiments have been deposited at Zenodo: https://zenodo.org/records/7327363 and are publicly accessible.
• The processed 10× Visium samples of the human coronary arteries (shown in the Figure S2), have been deposited at Zenodo: https://zenodo.org/records/14007461 and are publicly accessible.
• The code used in the analysis of datasets and generation of results for this study has been deposited at Zenodo: https://zenodo.org/records/16920741, and is publicly accessible.
• Any additional information required to reanalyze the data reported in this study is available from the lead contact upon request.

## Acknowledgments

This study was supported by grants from the 10.13039/501100009708Novo Nordisk Foundation (nos. NNF17OC0030688 and NNF20SA0061466 to J.F.B.), the 10.13039/501100000781European Research Council under the European Union’s Horizon 2020 research and innovation program (grant no. 866240 to J.F.B.), and the 10.13039/501100002739Aarhus University Research Foundation (Starting Grant, AUFF-E-201 9-7-23 to J.A-J.). The results of this manuscript are part of the Project THOR (Targeting smooth muscle cells in atherosclerosis therapy) of the Open Discovery Innovation Network (ODIN) initiative. Some schematics were made using Biorender.com.

## Author contributions

J.A-J. participated in the design of the experiments and performed *in vitro* experiments, analysis, interpretation of data, and generation of figures; A.M. performed the re-integration of scRNA-seq datasets and RNA-seq bioinformatics analysis and contributed to the generation of figures; A.L.J. performed *in vitro* experiments and analysis; P.L.M. performed the initial analysis on GWAS datasets and potential candidate genes; A.K.U. interpreted the data; D.D. contributed to the scRNA-seq analysis and selection of target genes; J.H. performed *in vitro* experiments and analysis; L.F.J. performed *in vitro* experiments and analysis; D.S. performed bioinformatics analysis in SCENIC; C.P. and J.M. performed the spatial transcriptomics analysis; G.B. interpreted the data; J.B. interpreted the data; K.H. interpreted the data; L.M.R. provided experimental assistance for *in vitro* experiments; M.T. performed the genetic analysis of directionality; M. Nyberg contributed in the conceptualization of the project and interpreted the data; M. Nyegaard conceptualized the project and interpreted the data; J.F.B. conceptualized the project, designed the experiments, and interpreted the data; J.A-J., A.M., and J.F.B. drafted the manuscript and figures with important contributions from A.L.J., P.L.M., A.K.U., J.H., L.F.J., D.S., C.P., G.B., J.B., K.H., J.M., M.T., M. Nyberg, and M. Nyegaard. All authors read and approved the manuscript.

## Declaration of interests

A.K.U., D.D., C.P., J.M., G.B., J.B., K.H., M.T., and M. Nyberg are employed at Novo Nordisk A/S.

## STAR★Methods

### Key resources table

**Table:** 

REAGENT or RESOURCE	SOURCE	IDENTIFIER
**Chemicals, peptides, and recombinant proteins**		

Cholesterol-Water Soluble	Sigma-Aldrich	Cat# C4951 Batch:0000427939
Human tumor necrosis factor-alpha (TNF) protein	Abcam	Cat# Ab259410
Human interferon alpha 1 (IFN) protein	Abcam	Cat# Ab48750
NucBlue™	Thermo Fisher	Cat# R37605
DAPI	Thermo Fisher	Cat# D1306
SYBR Green qPCR master mix	Thermo Fisher	Cat#K0252

**Critical commercial assays**		

Click Tech EdU cell proliferation kit 488	Sigma-Aldrich	Cat# BCK488
NucleoSpin RNA Plus Kit for RNA purification	Macherey-Nagel	Cat# 790984

**Deposited data**		

RNA-seq data from siRNA-guided target gene knockdown experiments	This paper	Zenodo: https://zenodo.org/records/7327363
Processed 10x Visium samples of the human coronary arteries (shown in the Figure S2)	Bleckwehl et al.^26^	Zenodo: https://zenodo.org/records/14007461
Code used in the analysis of datasets and generation of results for this study	This paper	Zenodo: https://zenodo.org/records/16920741
Human reference genome NCBI build 38, GRCh38	Genome Reference Consortium	http://www.ncbi.nlm.nih.gov/projects/genome/assembly/grc/human/

**Experimental models: cell lines**		

Human aortic smooth muscle cells	ATCC	Cat# PCS-100-012, Lot #80323179

**Oligonucleotides**		

siRNA targeting sequences, see Table S13	QIAGEN	N/A
Primer sequences, see Table S14	This paper	N/A

**Software and algorithms**		

Image J, version 2.14.0/1.54f	Schneider et al.^68^	https://imagej.net/ij/
NIS-Elements Imaging software 5.21.0.14830	Nikon	https://www.microscope.healthcare.nikon.com/en_EU/products/software/nis-elements
GraphPad Prism, version 9.5.1	GraphPad Software	https://www.graphpad.com/
Space Ranger, version 1.3.0	10X Genomics	https://www.10xgenomics.com/support/software/space-ranger/downloads
Cell Ranger, version 6.1.2	10X Genomics	https://www.10xgenomics.com/support/software/cell-ranger/downloads
R software, versions 4.1.1 and 4.2.3	R Core Team	https://www.R-project.org/
Seurat (R package), version 4.9.9.9	Stuart et al.^69^	https://satijalab.org/seurat/
Harmony (R package), version 1.0.1	Korsunsky et al.^70^	https://cran.r-project.org/package=harmony
PopV (R package), version 0.2	Ergen et al.^71^	https://github.com/YosefLab/popV
Bioconductor (R packages), version 3.14	Huber et al.^72^	https://www.bioconductor.org/
DESeq2 (R package), version 1.34.0	Love et al.^73^	https://bioconductor.org/packages/DESeq2/
BioNERO (R package), version 1.2.0	Almeida-Silva and Venancio^74^	https://www.bioconductor.org/packages/BioNERO/
RcisTarget (R package), version 1.14.0	Aibar et al.^75^	https://bioconductor.org/packages/RcisTarget/
SCENIC (R package), version 1.1.2	Aibar et al.^75^	https://scenic.aertslab.org/
WGCNA (R package), version 1.70	Langfelder and Horvath^76^	https://cran.r-project.org/package=WGCNA
Bisque (R package), version 1.0.5	Jew et al.^77^	https://github.com/cozygene/bisque
randomForest (R package), version 4.7-1.1	Liaw and Wiener.^78^	https://cran.r-project.org/package=randomForest
PLINK, version 1.9	Chang et al.^79^	https://www.cog-genomics.org/plink/
nf-core/rnaseq pipeline, version 3.5	Ewels et al.^80^	https://github.com/nf-core/rnaseq/
STAR aligner, version 2.6.1d	Dobin et al.^81^	https://github.com/alexdobin/STAR

**Other**		

Flexcell tension system	Flexcell	Cat# FX-5000 T
BioFlex 6-well culture plate	Flexcell	Cat# BF 3001P

### Experimental model and study participant details

#### Human aortic smooth muscle cells

We used healthy human aortic smooth muscle cells (haSMCs) from a single donor (male, 29 years, African American) (ATCC, PCS-100-012; Lot #80323179, LGC standards). SMCs were expanded at 37 °C, 5% CO_2_ in vascular cell basal medium with cell growth kit from ATCC (PCS100 030 and PCS 100 042) from passage P0 to passage P3. During cell expansion, plates were coated with type I collagen from rat tail (Corning, 354236) diluted in 0.1% acetic acid [15 μg/mL] for >20min at room temperature (RT) and washed once with phosphate-buffered saline (PBS). For experiments, SMCs of passage P4 and P5 were cultured in smooth muscle cell growth medium containing 5% fetal calf serum and supplements (Provitro, 211-0601).

#### Ethical statement

The scRNA-seq datasets were obtained from public studies involving explanted hearts of transplant recipients (for human coronary arteries) and carotid endarterectomies (for human carotid arteries). As stated elsewhere,^5^^,^^6^^,^^7^ all patients provided informed consent through forms approved by institutional review boards. The analysis involving human coronary arteries (spatial transcriptomics) was previously reported^25^ and approved by the National Videnskabsetisk Komité and the Regional Ethics Committee in Copenhagen, Denmark. Adhering to local legislation and institutional requirements. The human coronary samples used in spatial transcriptomics were acquired from a commercial supplier (AnaBios Corporation) of tissues donated by organ donors who had consented to donate tissues for research. Human arterial smooth muscle cells used for the *in vitro* analysis were obtained from a commercial supplier (American Type Culture Collection, ATCC), where donors signed an informed consent form in accordance with the World Medical Association Declaration of Helsinki on ethical principles for medical research involving human subjects.

### Method details

#### Mining of genome-wide association studies (GWAS) datasets

We focused on GWAS from the Cardiovascular Disease Knowledge Portal (CVDKP)^14^ and Finngen r5.^15^ Datasets were extracted from CVDKP using Signal Sifter (https://cvd.hugeamp.org/signalsifter.html). We searched for coronary artery disease (CAD) traits, excluding those with lipoproteins (LDL) associations. A ratio was calculated between log10(P value) of CAD/LDL. Hits were included if ratio > 2 and LDL P value > 5e-8. For Finngen datasets, credible sets and top hits were extracted using coronary atherosclerosis (“I9_CORATHER”) as a phenotype of interest, choosing the data tables “Traditional” (with a gene nearest to genomic variant) or “Credible Sets” (where genomic variant is fine mapped to a leading variant and related nearest gene). Genes were removed if already identified by the previous method, or LDL P value ≤ 5e-8 or co-association to lipoprotein traits were discovered.

#### Retrieving genes assigned to genomic variants

The primary list of genes assigned to the collected genomic variants was compiled as described above. Additionally, each genomic variant was checked using the results of “Variant-to-Gene” (V2G) and “Locus-to-Gene” (L2G) fine-mapping pipelines of the Open Targets Genetics platform.^16^ Evidence in V2G and L2G approaches is based on distance between the variant and the gene transcription start site, and the data from several types of experiments, including molecular phenotype quantitative trait loci (QTL), chromatin interaction, *in silico* functional prediction. A detailed description of these approaches can be found in the documentation of the Open Targets Genetics platform. If any additional gene was identified, it was added to the list.

#### Integration of single-cell RNA sequencing studies from public datasets

To estimate the transcriptomic patterns of target genes and gene networks in different cell types, we collected scRNA-seq data of human atherosclerotic lesions and healthy arteries published previously.^5^^,^^6^^,^^7^ Sample information was gathered from the Gene Expression Omnibus database (accession GSE159677, GSE155512 and GSE131778). Raw FASTQ files were downloaded from NIH Sequence Read Archive (SRA) and processed via Cell Ranger version 6.1.2 with 10X Genomics-derived human reference hg38 (2020-A July 7, 2020). Single-cell data filtering, integration, and clustering were done in the Seurat R package.^69^ Cells were filtered as follows: number of UMIs > 500, number of detected genes between 200 and 4500, percent of mitochondrial genes < 10% and hemoglobin genes < 1%, ratio of mitochondrial to ribosomal genes < 0.5.^82^ Additionally, we discarded the barcodes suspected as doublets using any of the three methods: DoubletFinder,^83^ scDblFinder,^84^ and Scrublet.^85^

We normalized data using the standard “LogNorm” method in Seurat, and integrated datasets using Harmony.^70^ Cell clusters were identified using a shared nearest neighbor modularity optimization-based algorithm (implemented in Seurat by default) with 2000 most variable genes, 30 principal components, and a resolution of 1. Marker genes of the identified cell clusters were found with Wilcoxon–Mann–Whitney test implemented in Seurat. Automatic cell type annotation was performed using the popV pipeline with the Tabula Sapiens “Vasculature” dataset as reference.^71^ To validate the SMC-specific origin of identified clusters, we integrated human data with a scRNA-seq dataset of SMC-derived cell population from *Myh11*-CreER^T2^ x TdTomato (TdT) reporter mice with induced atherosclerosis and high-fat diet as described elsewhere.^8^ Cell clusters within the SMC-containing mesenchymal supercluster were further re-annotated based on marker genes (differentially expressed in a specific cell cluster compared to other cell clusters). Amongst the mesenchymal cells, one cluster called “undefined” contained low-quality droplets that passed the filtration step but had dramatically lower numbers of UMIs and detected genes than other cells. This cluster may represent the fragments of mesenchymal cells and, thus, was not considered for further analysis.

#### Selection of smooth muscle cell population-specific genes

According to scRNA-seq results, several clusters of SMCs were included in a unified supercluster of “mesenchymal cells.” Since a clear delineation between the spectrum of differentiated and modulated SMCs and other transcriptionally close cell types, including fibroblasts and pericytes, remains challenging, we consider the whole mesenchymal supercluster to determine the SMC specificity of each gene. We defined a gene as a mesenchymal marker and considered as a candidate if it was significantly more expressed in mesenchymal cells (P value after Bonferroni correction <0.05, log2 fold change ≥ 0.5) and detected in at least 20% more cells in the mesenchymal supercluster than in the set of other cells.

#### Spatial transcriptomics

Spatial transcriptomics samples were generated from formalin-fixed and paraffin-embedded (FFPE) human coronary arteries at different stages of the disease, isolated from explanted hearts, as previously described.^25^^,^^26^ Briefly, 5μm thickness sections were mounted on Visium slides and processed for spatial transcriptomics according to the 10X Genomics Visium FFPE Version 1 protocol. Samples were deparaffinized, stained with hematoxylin and eosin (H&E), and imaged using VS200 Slide Scanner (Olympus Life Science) before decrosslinking, destaining and overnight probe hybridization with the 10X Visium Human version 1 probe set. The following day, hybridized probes were released from the tissue and ligated to spatially barcoded oligonucleotides on the Visium Gene expression slide. Barcoded ligation products were then amplified and used for the construction of libraries, which consequently were sequenced on a NovaSeq 6000 sequencing platform (Illumina) using a NovaSeq 6000 S2 Reagent Kit v1.5 (Illumina) according to the manufacturer’s instructions. Afterwards, reads were aligned with their corresponding probe sequences, mapped to the Visium spot where each probe was captured, and finally aligned with the original H&E-stained image of the tissue section using the software Space Ranger version 1.3.0 (10X Genomics). Samples ranged from 568 to 1,746 barcoded 55-μm spots. 1563 genes were identified per spot on average. Each spot represents a mixture of cells, typically between 5-10 cells. Quality control of the Visum slides showing the total counts and the genes for each specimen were previously reported.^26^

#### Small interference RNA (siRNA) transfection

For a subset of genes, we compared the effects of individual small interfering RNAs (siRNAs) to a set of two pooled siRNAs. While some individual siRNAs produced effects similar to the pooled set, in other cases, the pooled siRNAs were more effective. Based on these observations and to ensure consistency across targets, all downstream experiments were conducted using two pooled siRNAs per target gene (QIAGEN, 20 μM). All siRNA sequences are shown in Table S13. All the siRNAs utilized in this study were screened by the manufacturer for various sequence motifs (e.g., TLR7), known to trigger an interferon or cytokine response. Consequently, siRNAs containing these motifs were used in our study. Transfection was performed with Opti-MEM (Thermo Fisher, Cat. No. 31985062) and Lipofectamine RNAiMAX (Invitrogen, Cat. No. 13778150) on two consecutive days according to the manufacturer's instructions. Control SMCs were transfected with control siRNA (QIAGEN, AllStars Negative S103650318, 20 μM).

#### Baseline modulation of human smooth muscle cells

Human SMCs were cultured on type I collagen-coated plastic 12-well plates with growth medium as described above. Cells cultured under these conditions spontaneously modulated their phenotype and thus were used as a “baseline.” When the cells reached 70% confluency, they were transfected with siRNAs for each gene on two consecutive days, as described above. Every RNA sample was a pool of three wells, and we used four technical replicates per condition. Analysis was performed one day after the second transfection.

#### Cholesterol overload

Human SMCs were cultured on type I collagen-coated plastic 12-well plates with growth medium. At 70% of confluency, cells were either untreated or treated for 72 hours (h) with water-soluble cholesterol (Sigma-Aldrich, C4951, batch 0000427939, final concentration 10 μg/mL). For initial RNA-seq analysis, we compared expression levels of SMCs treated with cholesterol overload for 72 h versus untreated cells. Cells were stained with Oil red O (ORO) solution (5 mg/mL) for 10 min following by haematoxylin (HE) counterstain for 5 min. For target gene characterization, SMCs were treated with cholesterol and transfected in parallel with siRNAs for each gene on two consecutive days while continuing cholesterol treatment for another day to reach 72 h in total. Every RNA sample was a pool of three wells, and we used four technical replicates per condition.

#### Mechanical stretch

Human SMCs were seeded onto flexible silicon 6-well plates coated with ProNectin (BioFlex, Cat. No. BF 3001P) and cultured in growth medium until 70% confluency. For initial RNA-seq analysis, we compared expression levels of SMCs subjected to stretch (10% cyclic stretch with a sinusoidal waveform at a frequency of 1 Hz for 6 h) with the Flexcell tension system (Flexcell International Corp., FX-5000 T) versus static conditions. For target gene characterization, SMCs were transfected with siRNAs for each gene on two consecutive days. A day later, control and target transfected cells were subjected to stretch. Every RNA sample was a pool of two wells, and we used four technical replicates per condition. Data was evaluated by RNA-seq as described above.

#### TNF and IFN treatment

Stimulation with tumor necrosis factor-alpha (TNF) or interferon-alpha (IFN) was performed for 24h when SMCs reached a confluency of ∼80-90% (approximately 48 h after seeding). SMCs were cultured on type I collagen-coated plastic 12-well plates and stimulated with human TNF (Abcam, ab259410, 10 ng/mL), human IFN (Abcam, ab48750, 100 ng/mL), or left untreated. Every RNA sample was a pool of three wells (12-well plates), and we used four technical replicates per condition.

#### Cell proliferation

A total of 50,000 human SMCs were seeded on glass coverslips coated with type I collagen in a 24-well plate. One day after the second transfection, cells were then cultured for an additional 24 h in growth medium containing 5-ethynyl-2'-deoxyuridine (EdU,10 μM). Cells were fixed in 3.7% paraformaldehyde in PBS for 15 min. The EdU-positive cells were labeled according to the manufacturer’s instructions (Sigma-Aldrich, BCK488), followed by nuclei labeling with DAPI (Thermo Fisher, D1306). Coverslips were mounted on slides and imaged using a Nikon Eclipse Ti2 inverted microscope. The NIS-Elements software was used for image acquisition, and ImageJ^68^ was used for quantification. Three to four technical replicates were evaluated per gene.

#### Scratch assay for migration

Human SMCs were seeded in 24-well plates coated with type I collagen and transfected with siRNAs as described above. One day after the second transfection, the confluent cell monolayer was scratched in the center of the well using a 10 μl pipette tip before washing once with PBS to clear cell debris and floating cells. Cells received fresh media, and the nuclei were labeled with Hoechst 33342 live probe (NucBlue™, Thermo Fisher R37605). Time-lapse images were captured hourly for 24 h using a Nikon Eclipse Ti2 inverted microscope with an integrated chamber ensuring proper cell culture conditions of 37 °C and 5% CO_2_. The migration ability was measured by calculating the percentage of gap closure at different time points compared to time 0 (baseline) using the NIS-Elements software. Three to four technical replicates were evaluated per gene.

#### Total mRNA sequencing

RNA was isolated from wells using the NucleoSpin RNA Plus Kit (Macherey-Nagel, 790984) and sequenced by BGI (Copenhagen, Denmark). All RNA samples passed quality control (Agilent 4200 electrophoresis system). Non-stranded cDNA libraries were prepared with polyA-selected mRNA, and 100 bp paired-end sequencing was conducted with at least 20M pair reads per sample using DNBSEQ. RNA sequencing (RNA-seq) data were processed with the Nextflow-based pipeline “nf-core/rnaseq” (version 3.5).^80^ Sequenced reads were aligned to the GRCh38 human reference genome using STAR aligner (version 2.6.1d),^81^ and gene-level quantification was done with Salmon (version 1.5.2). The reference genome sequence and gene annotation were downloaded from AWS iGenome using the Ensembl version as a source. Further analysis was performed in R (version 4.1.1) with Bioconductor (version 3.14) packages.^72^ Gene counts were imported using tximport. Gene filtering was performed as previously described, and only genes with ≥10 counts per million (CPM) in at least 3 samples were analysed. DESeq2^73^ was used for data normalization, variance stabilizing transformation, principal component analysis, and estimation of differential gene expression between compared groups. Estimated P values (Padj) were adjusted for false discovery rate by Benjamini and Hochberg procedure.^86^ Genes with Padj <0.05 and absolute log2 fold change (LFC) ≥0.5 were considered differentially expressed, or significantly dysregulated. To identify involved pathways, the lists of differentially expressed genes were used for gene-set enrichment analysis using a Web-based gene set analysis toolkit (WebGestalt)^87^ with categories from GeneOntology,^88^ Reactome,^34^ WikiPathways,^35^ and KEGG Pathway^33^ databases.

#### Real time-qPCR

The relative gene expression was measured by real-time qPCR (RT-qPCR) using *HPRT1* as housekeeping gene. Intron-spanning primers amplifying 50<150bp amplicons were designed using the NCBI Primer-BLAST.^89^ Primer efficiency was tested by plotting the sample quantity of the dilution series to the average Ct values of each dilution on a logarithmic scale. The primer sequences are shown in Table S14. The Maxima SYBR Green qPCR master mix kit [1:2] including ROX [0.18μM] (Thermo Fisher Scientific, K0252) was mixed with forward and reverse primers [268 nM] and cDNA [12.5 ng] in a final volume of 14 μL. On a CFX Opus Biorad machine, the RT-qPCR program was run with an initial denaturation step of 95°C for 10 min followed by 40 cycles of 95°C for 30 sec, 60°C for 1 min, and 72°C for 1 min, and one cycle of 95°C for 1 min, 60°C for 30 sec, and 95°C for 30 sec.

#### Regulon enrichment analysis

The lists of unidirectionally dysregulated genes were screened for the enrichment of transcription factor binding motifs using Bioconductor^72^ R package RcisTarget.^75^ A set of identified motifs and related transcription factors from TRANSFAC^90^ database collection was used for further regulon analysis. Regulon activity analysis in the integrated scRNA-seq dataset data of human atherosclerotic lesions and healthy arteries^5^^,^^6^^,^^7^ was performed using the SCENIC pipeline^75^ with default parameterization and human genome reference (hg38). Area under the curve (AUC) values estimated by the pipeline were averaged for every cell cluster and z-scaled by regulon. Regulons with higher relative activity (positive z-score) in the cluster of interest were considered active.

#### Genetic direction of selected CAD-associated genes

We searched for annotated CAD GWAS loci^13^ and performed co-localization and Mendelian Randomization using e/pQTLs collected from several sources (GTEx V8,^42^ eQTLgen;^43^ eQTL catalogue;^91^ UK Biobank;^44^ and deCODE genetics^45^) for selected target genes. Co-localization analysis was performed using HyPrColoc^92^ with a posterior probability of alignment threshold of 0.7. For co-localized e/pQTLs, we determined the impact on CAD risk using Mendelian randomization with the e/pQTL (p<5e-6) as the instrumental variable and the CAD GWAS as the outcome. We determined independent genetic associations for the e/pQTL using LD-clumping in PLINK (version 1.9), based on an r2 threshold of 0.01 within a 500kb region around the gene.^79^ When the e/pQTLinstrument was composed of a single genetic variant, we used the Wald ratio approach to derive the estimate, while when multiple SNPs were involved, we used the inverse variance weighted approach.^93^

#### Gene co-expression network inference

We inferred the control gene co-expression network (GCN) using the results of RNA-seq for human SMCs transfected with control siRNA in every cellular assay. Briefly, we employed a gene expression matrix after variance stabilizing transformation. We selected the top 5000 most variable genes for weighted gene co-expression network analysis (WGCNA)^76^ in BioNERO R package.^74^ Seven of the 94 control samples were identified as outliers by the standardized connectivity method^94^ and discarded for further analysis. We used Spearman’s correlation to build a signed network with the power soft threshold 7, achieving scale-free topology R^2^=0.843 (scale-free topology and mean connectivity for different power thresholds are shown in Figure S9). Correlation analysis between module eigengene (the first principal component of the gene expression matrix of the corresponding module) and cellular assay was used to identify gene modules responsive to baseline modulation, cholesterol overload, or mechanical stretch. Noteworthy, we did not adjust RNA-seq data to correct the difference between experimental batches before GCN inference since they are compromised by study design with a cellular state. Instead, we considered modules with clear patterns of higher differences between cellular states than within-state variation. Over-representation analysis of genes assigned to different GCN modules in Reactome, KEGG Pathway, WikiPathways, GeneOntology, and Broad Institute Hallmark50 gene categories was performed with WebGestalt, as previously described (Table S12).

#### Cell type deconvolution

We conducted cell-type deconvolution analysis to predict which mesenchymal cell states observed *in vivo* in atherosclerotic lesions can be mirrored by SMCs cultured *in vitro* in our experiments. Transcriptional profiles of mesenchymal cell clusters identified in the integrated scRNA-seq datasets – including contractile, transitional, osteochondrogenic, and pericyte-like SMCs, as well as fibromyocytes and fibroblasts – were utilized as a reference. We estimated the relative proportions of these clusters based on cell counts and generated pseudo-bulk data by summarizing gene counts (using the *AggregateExpression* function in Seurat) in mesenchymal cell supercluster per sample. Then we estimated the relative proportions of mesenchymal cell components in bulk RNA-seq samples from our *in vitro* experiments using the method Bisque.^77^ This method employs scRNA-seq data as a reference to retrieve gene expression profiles and proportions for the given cell type annotation and to learn gene-specific bulk transformations, and then cell proportions from bulk RNA-seq data can be estimated with non-negative least-squares regression. To check the precision of this method, we also predicted relative proportions in pseudo-bulk samples based on scRNA-seq data and compared them with ground-truth proportions (Figure S3). We compared separate component proportions between groups of samples using the Wilcoxon–Mann–Whitney test. P values estimated for multiple comparisons were adjusted by the Bonferroni–Holm method.

#### Gene signature scoring

We performed gene expression-based scoring of regulation for those gene signatures which, to our knowledge, are related to SMC contractility, adhesion, and cell cycle, as well as with synthesis of components of extracellular matrix and inflammatory signaling. Specific gene sets were collected from the pathway databases: KEGG Pathway,^33^ Reactome,^34^ and WikiPathways^35^ (Table S6). Additionally, we used top up- and downregulated genes after cholesterol loading, stretch, TNF, or IFN stimulation of SMCs as gene signatures (Padj <0.05, absolute LFC ≥ 0.5, arranged by LFC, and top 100 genes are taken). We also used the gene list for every GCN module as gene signature (for large modules, only 100 genes with the highest intramodular connectivity were chosen). Gene signature scoring was conducted with a single-sample scoring method, singscore.^95^ This rank-based approach allowed us to score changes in transcript abundance between target knockdown and related control groups for every gene signature of interest. To accomplish this, Wald test z-statistics produced by DESeq2 after comparison between relevant groups of samples was used for gene ranking instead of conventional gene expression matrix. Relative scores for selected gene signatures were estimated for every comparison of target knockdown versus related control in all studied cellular assays. Thus, the estimated z-scores reflect the change in total transcriptional activity for every gene signature.

We assessed the ability of GCN module-specific gene signatures to predict the effect of target genes with identified genetic direction on CAD. Activity score of each GCN module was calculated for every target knockdown and assay, as described above. Random forest model (implemented in the randomForest package^78^) was trained with gene module scores as explanatory variables and the effect of target gene knockdown on CAD as an instrumental variable. The embedded measure of node impurity, mean decrease in Gini index, was used to assess how important the contribution of each variable was in the final model prediction.

### Quantification and statistical analysis

All experiments were conducted independently in three to four replicates. GraphPad Prism 9.5.2 Software (GraphPad Software, Inc., San Diego, CA, USA) and R software, version 4.1.1 (R Core Team) were used for graphical representation and statistical analysis. Quantification and specific statistical test for each experiment and analysis are described in method details, respectively. The statistical detail of the experiments are also presented in the figure legends.

Data are represented as mean ± SEM or as log2-transformed fold change (LFC) of average gene expression; error bars are LFC standard error. Statistical significance was defined as follows, adjusted p value (Padj): ∗ <0.05, ∗∗ <0.01; ∗∗∗ <0.001, ∗∗∗∗ <0.0001 or ns. (not significant).

## Supplemental information

Document S1. Figures S1–S10

Table S1. Target list of CAD genes identified after LDL-cholesterol exclusion, related to Figure 1

Table S2. Genes upregulated in every identified cell cluster compared to other cells of integrated plaque scRNA-seq data, related to Figure 1

Table S3. List of target genes with methods used for selection, related to Figure 1

Table S4. Gene expression comparing cholesterol overload and stretch versus untreated SMCs, related to Figure 2

Table S5. Absolute expression levels of candidate genes in SMCs at baseline or after knockdown, related to Figure 2

Table S6. Gene set enrichment analysis of differentially expressed genes, related to Figure 3

Table S7. Gene signatures used to score relevant pathways in smooth muscle cells, related to Figure 3

Table S8. TNF- and IFN-regulated genes, related to Figure 3

Table S9. Regulons up- and downregulated per condition, related to Figure 4

Table S10. Target gene directionality, related to Figure 6

Table S11. Modules of gene correlation network, related to Figure 6

Table S12. Enrichment analysis of genes with the highest intramodular connectivity (up to 100 genes) in the modules of gene co-expression network, related to Figure 6

Table S13. Sequences of small interfering RNA used for target gene knockdown, related to STAR Methods

Table S14. Human primer sequences used for RT-qPCR, related to STAR Methods
