# Supplementary material for: Mapping atherogenesis mechanisms in smooth muscle cells by targeting genes linked to coronary artery disease
Source: iScience. 2025 Oct 4;28(11):113698. doi: 10.1016/j.isci.2025.113698 (PMC12589888; doi:10.1016/j.isci.2025.113698)
Supplement: Document S1. Figures S1–S10 [file mmc1.pdf]

iScience, Volume *28*

Supplemental information

Mapping atherogenesis mechanisms in smooth muscle cells by targeting genes linked to coronary artery disease

Julián Albarrán-Juárez, Anton Markov, Anne Louise Jensen, Peter Loof Møller, Anna Katarzyna Uryga, Djordje Djordjevic, Jakob Hansen, Lise Filt Jensen, Diana Sharysh, Charles Pyke, Jaime Moreno, Giulia Borghetti, Julian Bachmann, Kate Herum, Lisa Maria Røge, Matthew Traylor, Michael Nyberg, Mette Nyegaard, and Jacob Fog Bentzon

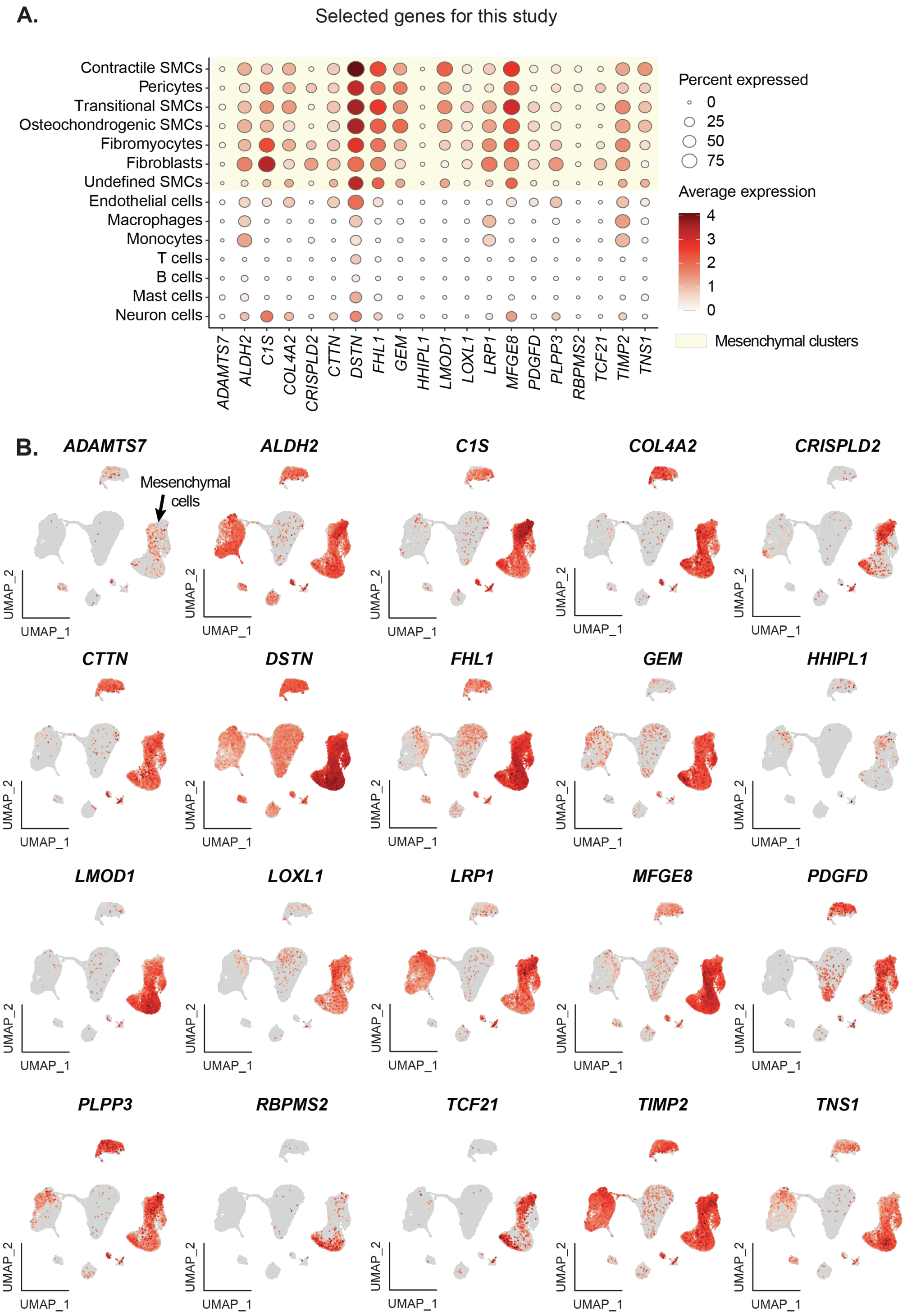

Figure S1: Expression of genes selected for the study in human atherosclerotic plaques. Related to Figure 1. A. Dot plot shows the expression of genes enriched in the mesenchymal supercluster (25456 cells) and selected for further study. Normalized and log2-transformed gene expression values are averaged for every cell cluster. **B.** UMAP plots of the selected target genes as analyzed by integrated public scRNA-seq data (50390 cells) on human atherosclerotic lesions. Scale is based on log2-transformed gene expression as shown in A.

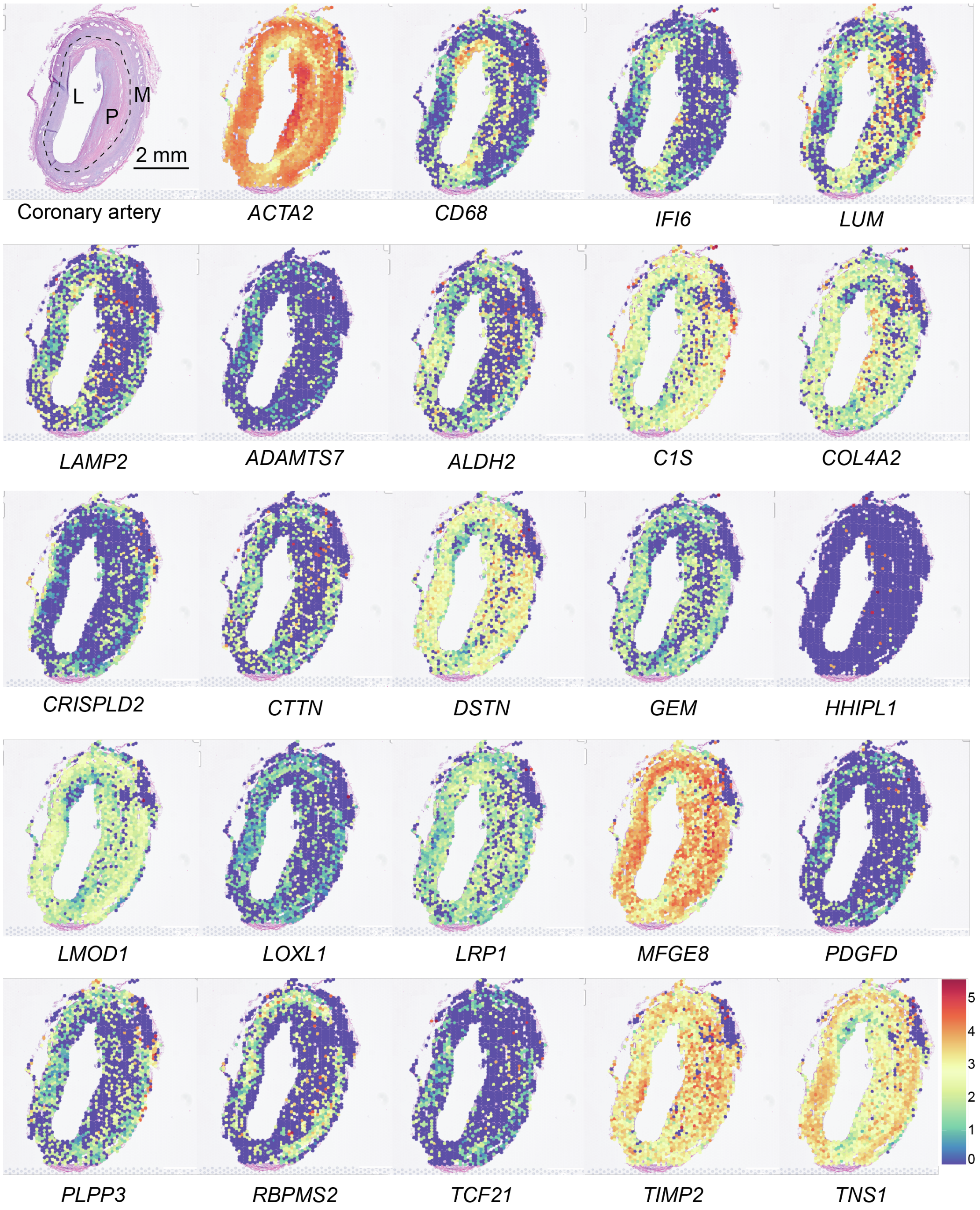

Figure S2: Spatial transcriptomics of selected genes in the study of human coronary arteries with atherosclerotic plaque. Related to Figure 1. A representative coronary artery atheroma stained with hematoxylin and eosin is shown (top left). Abbreviations: M (media), P (plaque), and L (lumen). Selected markers are shown to identify cell populations (smooth muscle cells; *ACTA2*, macrophages; *CD68*, inflammatory; *IFI6*, modulated smooth muscle cells; *LUM*, and macrophage-like *LAMP2*). Visium barcoded spots represent a mixture of 5 and 10 cells as originally showed by Bleckwehl et al 2025[26]. Shown is the representative section 8 that contains 1746 spots. Expression intensity is designated by the color scale that represents log2 normalized expression. *FHL1* was not captured in the panel and therefore no expression data is shown.

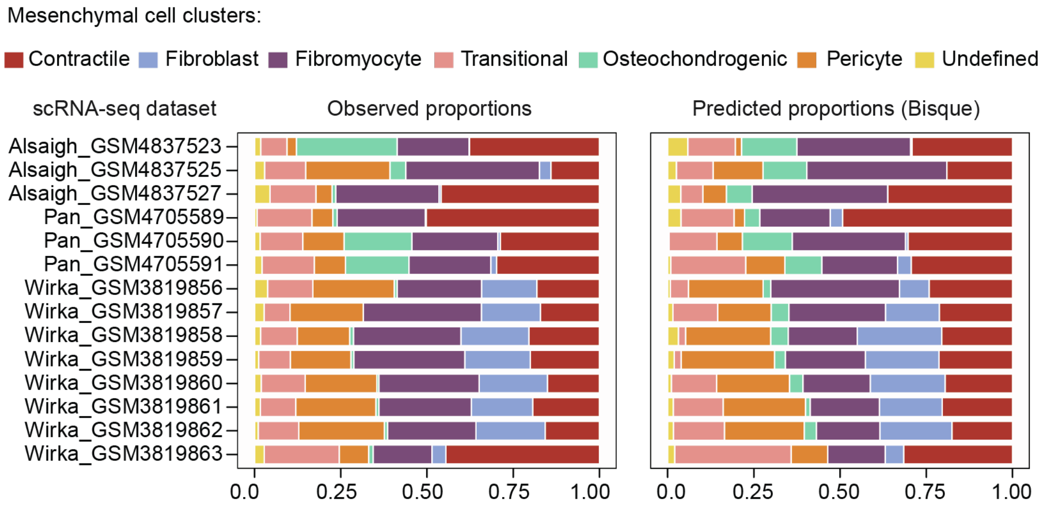

Figure S3: Mesenchymal cell type deconvolution. Related to Figure 2. Proportions of mesenchymal cell clusters observed in scRNA-seq data of human atherosclerotic plaques (left panel) and proportions predicted by cell type deconvolution of pseudo-bulk RNA-seq data based on by-sample aggregation of gene counts across mesenchymal clusters of the same scRNA-seq dataset (right panel).

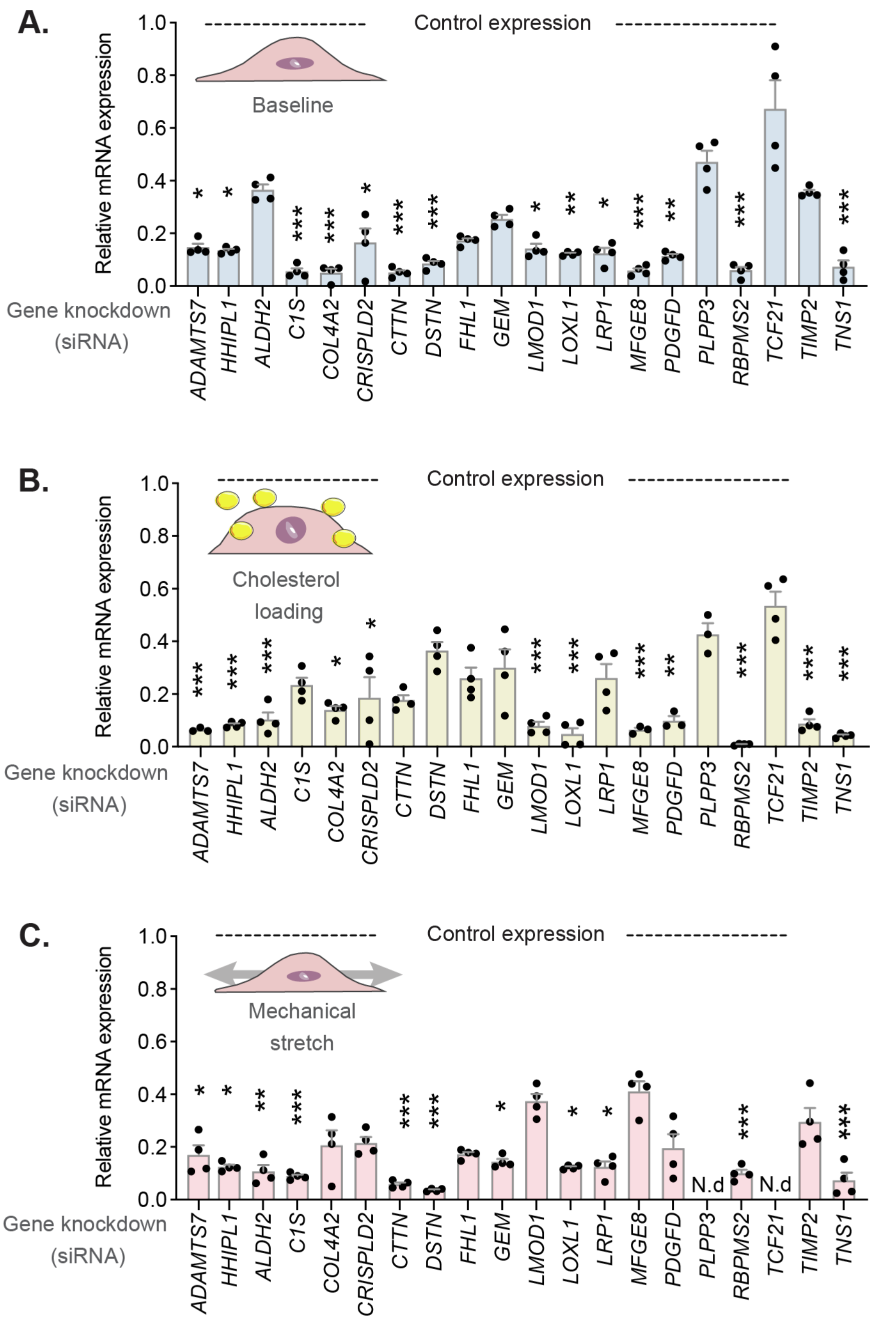

Figure S4: Validation of gene knockdown by real-time qPCR. **Related to Figure 2.** The knockdown efficiency of target genes in SMCs was evaluated by real-time qPCR under baseline (**A**), cholesterol loading (**B**), and mechanical stretch (**C**) conditions. Gene expression was normalized to the *HPRT1* housekeeping gene (control expression). For *TCF21* and *PLPP3,* the designed siRNAs reduced the expression by less than 50%. Therefore, *TCF21* and *PLPP3* were not further analyzed in the study. As indicated by N.d (gene expression was not determined). **(A-C)** Four technical replicates were tested for each gene. Data are presented as mean ± SEM. * Adjusted P value (Padj) < 0.05, ** Padj < 0.01; and *** Padj < 0.001 compared to control. Comparisons were analyzed by the Kruskal-Wallis test followed by Dunn's test with a control of false discovery rate by Benjamini and Hochberg method.

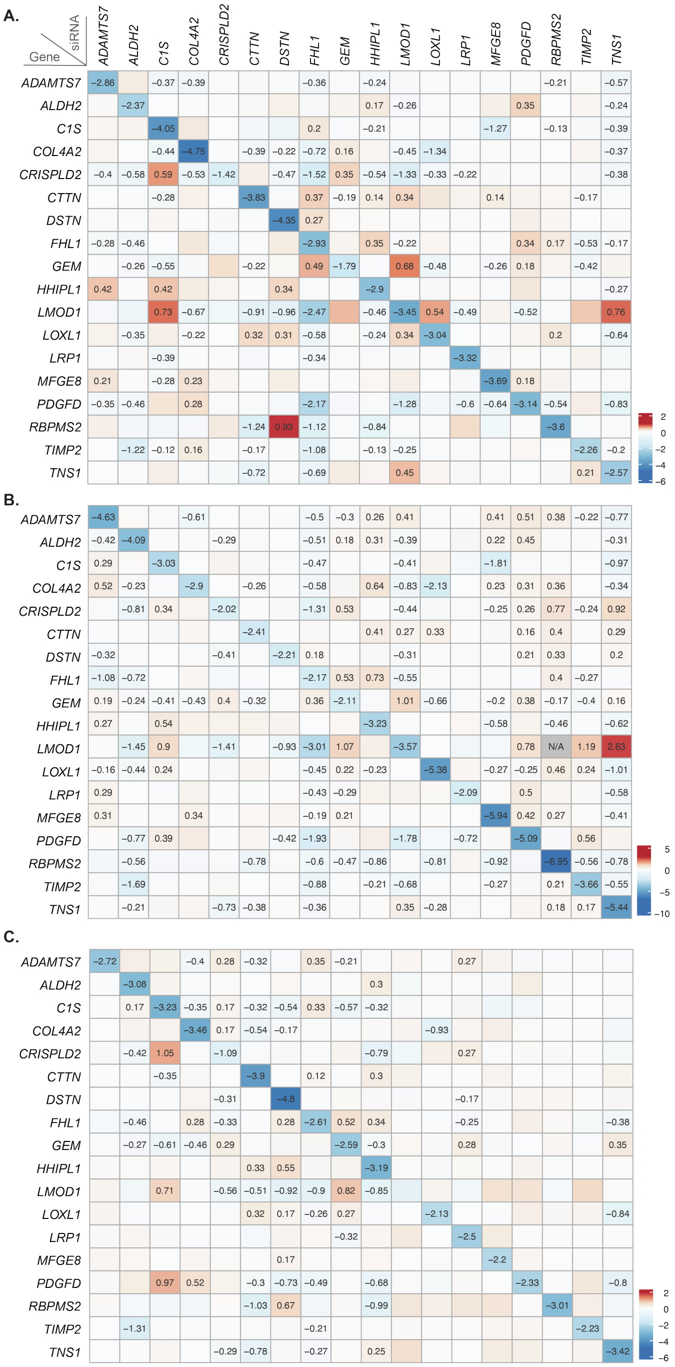

Figure S5. Knockdown efficiency of target genes by RNA-seq. Related to Figure 2. The knockdown efficiency of target genes was evaluated by RNA-seq analysis after baseline (**A**), cholesterol overloading (**B**), and mechanical stretch (**C**) conditions (3 or 4 technical replicates were used for every group of comparison). Genes that are knocked down with siRNAs are located on the X-axis. The measured gene expression is on the Y-axis. Scale is based on log2 transformed fold-change values, and numbers are shown for only significant results with an adjusted P value Padj < 0.05. N/A, indicates that the values are not available.

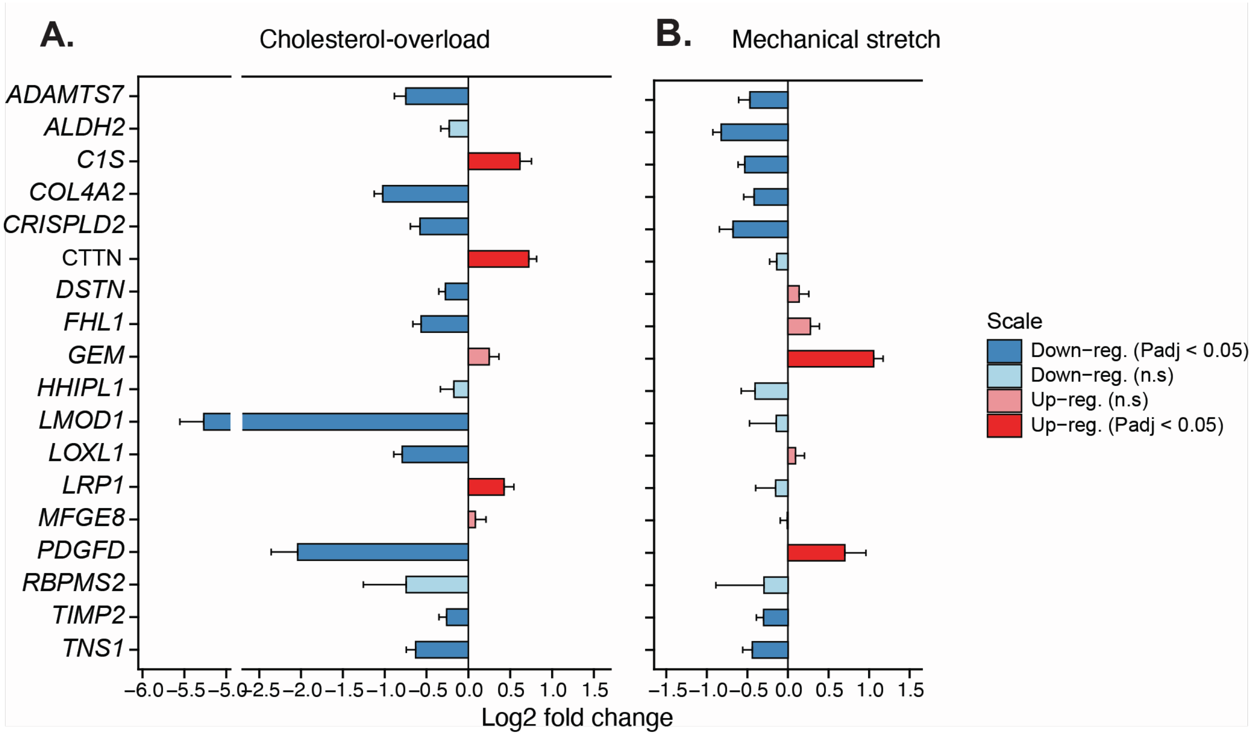

Figure S6: Regulation of target genes in different cellular assays. Related to Figure 3. Differences in target gene expression (RNA-seq) in cholesterol overload (**A**) and mechanical stretch (**B**) assays compared to baseline (3 technical replicates in each group). Bars represent log2-transformed fold change (LFC) of average gene expression; error bars are LFC standard error. The color scale indicates the direction of fold change (up- and down-regulated genes), significant results (Adjusted P value (Padj) for false discovery rate < 0.05) are highlighted with a saturated color. n.s indicates no significant values.

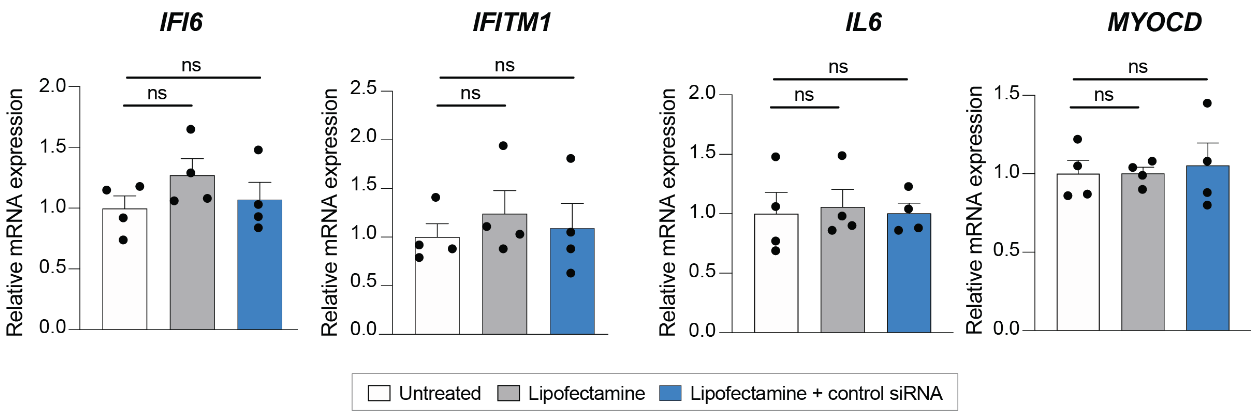

Figure S7: Effect of lipofectamine and control siRNA *per se* in knockdown experiments. Related to Figure 3. Shown is the expression of interferon-regulated genes (*IFI6, IFITM1*), TNF-regulated (*IL6*), or a prototypical contractile gene marker (*MYOCD*) in human SMCs treated with lipofectamine, control siRNA or left untreated. Gene expression was normalized to the housekeeping gene *HPRT1*. Four technical replicates were tested for each gene. Data are presented as mean ± SEM. Statistical significance was tested by one-way ANOVA followed by Bonferroni post hoc test. Non-significant results (adjusted P value (Padj) > 0.05) are shown as ns.

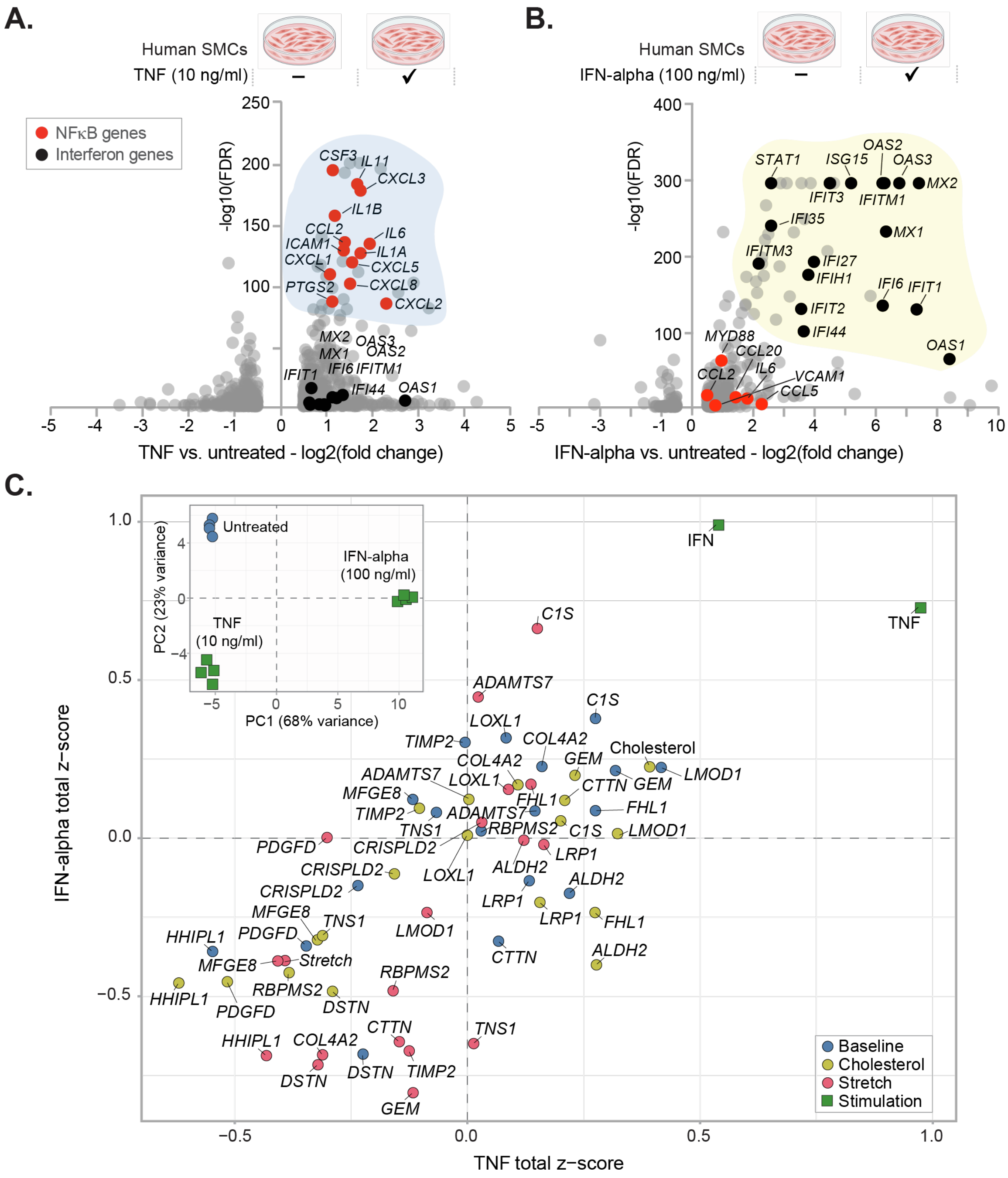

Figure S8: NFκB and type I interferon gene signatures in human SMCs. Related to Figure 3. Transcriptomic changes in human SMCs treated with **(A)** tumor necrosis factor (TNF, 10 ng/ml) and with **(B)** interferon alpha (IFN-alpha, 100 ng/ml) for 24h compared to untreated cells (4 technical replicates in compared groups). Shown in A and B are DEGs with an adjusted P value (Padj) < 0.05 and absolute log2 fold-change ≥ 0.5. DESeq2 was used for data normalization. **C.** To understand how NFκB and type I interferon gene networks are regulated by selected CAD genes, we scored the alterations in the expression of TNF and IFN-alpha gene signatures in every target gene knockdown and cellular assay (baseline, cholesterol overload, or stretch). The scores of the TNF and IFN-alpha stimulated cells compared to unstimulated control are added for reference (green square symbols). The inserted plot in **C** shows the principal component analysis (PCA) of cells stimulated with TNF, IFN-alpha, or left untreated. Cell culture elements in A and B were made with BioRender.com

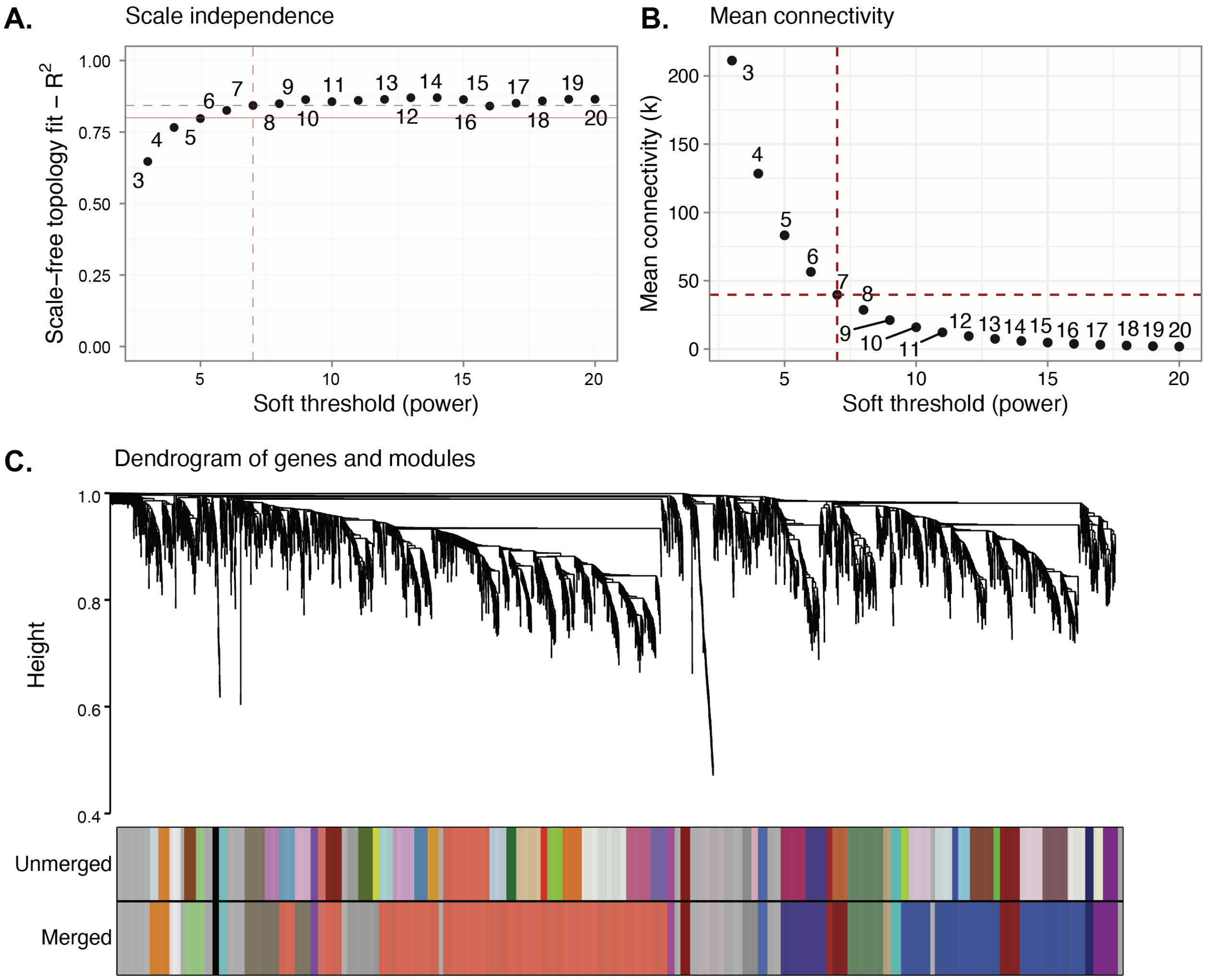

Figure S9: Gene network analysis in control human aortic smooth muscle cells. Related to Figure 6. A and **B** show the soft threshold power analysis results to ensure an optimal scale-free model fit index and mean connectivity, respectively, for weighted correlation network analysis of 94 samples of control siRNA-treated cells in our study. The red solid line demarcates a lower cutoff for the fit index (R^2=0.8). Dashed lines highlight the selected soft threshold**. C.** Dendrogram plot of 5000 genes in a co-expression network clustered based on a dissimilarity measure and their assignment to different modules (shown as different colors).

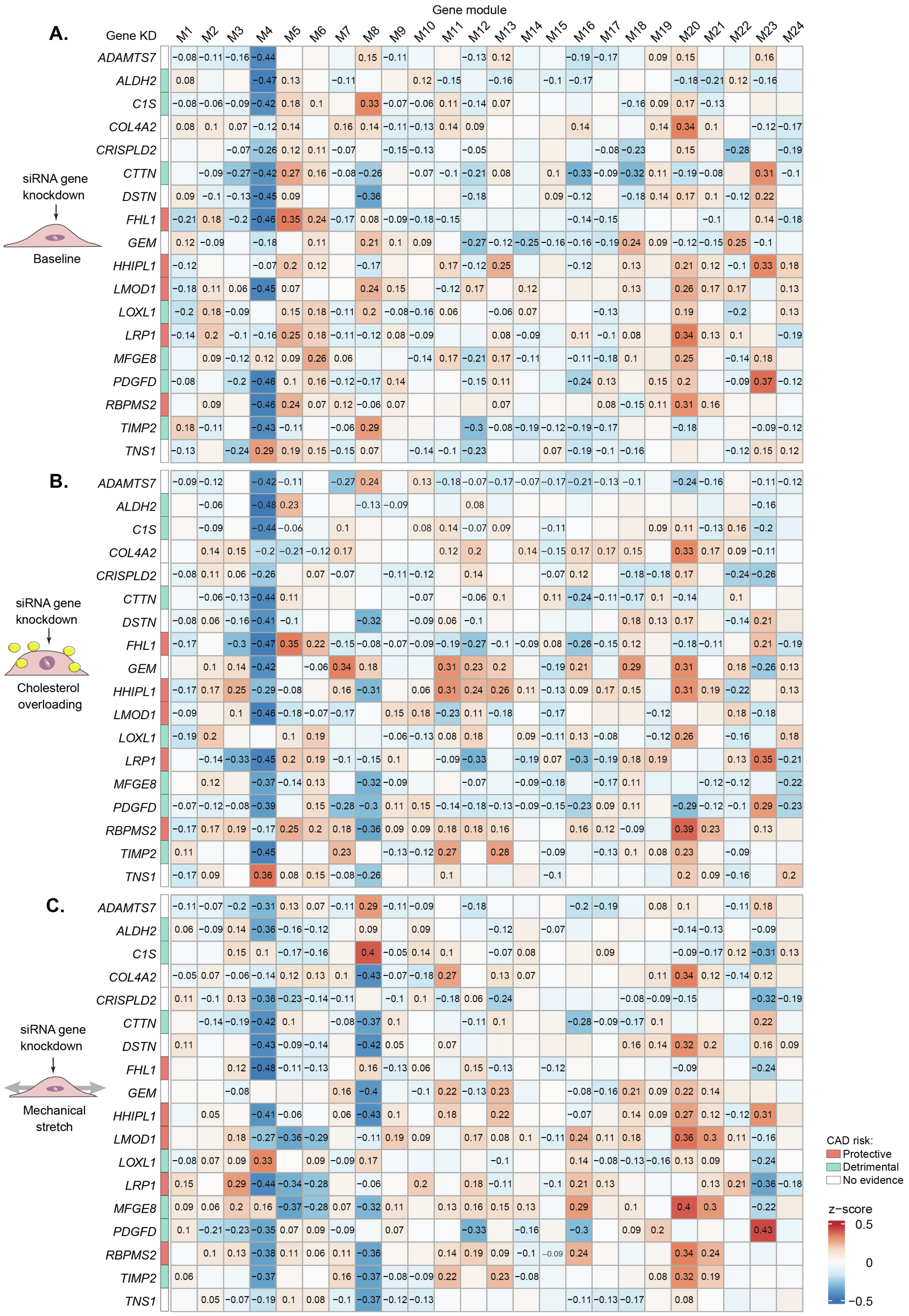

Figure S10. Gene modules co-expressed in human SMCs and disturbed after target gene knockdown. Related to Figure 6. Expression-based scoring of gene signatures for SMC-specific modules of co-expressed genes in the different cellular assays: **A.** baseline, **B.** cholesterol overload, and **C.** mechanical stretch. For gene modules 1 to 10, only the top 100 genes with the highest intramodular connectivity are considered for this figure. The color scale represents a z-score of deviation in the transcriptional activity of the relevant module's gene signature in target knockdown compared with a control group (3 or 4 technical replicates in each group). Only statistically significant z-score values are shown. Genetic direction is shown for genes associated with a decreased risk for coronary artery disease (CAD, salmon color), increased (emerald color), or unknown (grey color). Gene knockdown (KD).
